# NUB1 traps unfolded FAT10 for ubiquitin-independent degradation by the 26S proteasome

**DOI:** 10.1038/s41594-025-01527-3

**Published:** 2025-04-11

**Authors:** Connor Arkinson, Ken C. Dong, Christine L. Gee, Shawn M. Costello, Aimee Chi Soe, Greg L. Hura, Susan Marqusee, Andreas Martin

**Affiliations:** 1 California Institute for Quantitative Biosciences, University of California, Berkeley, Berkeley, CA USA; 2 Department of Molecular and Cell Biology, University of California, Berkeley, Berkeley, CA USA; 3 Howard Hughes Medical Institute, University of California, Berkeley, Berkeley, CA USA; 4 Biophysics Graduate Program, University of California, Berkeley, Berkeley, CA USA; 5 Molecular Biophysics and Integrated Bioimaging Division, Lawrence Berkeley National Laboratory, Berkeley, CA USA; 6 Department of Chemistry and Biochemistry, University of California, Santa Cruz, Santa Cruz, CA USA; 7 Department of Chemistry, University of California, Berkeley, Berkeley, CA USA; 8 Chan Zuckerberg Biohub, San Francisco, CA USA

**Keywords:** Enzyme mechanisms, Proteasome, Cryoelectron microscopy, SAXS

## Abstract

The ubiquitin-like modifier FAT10 targets hundreds of proteins in the mammalian immune system to the 26S proteasome for degradation. This degradation pathway requires the cofactor NUB1, yet the underlying mechanisms remain unknown. Here, we reconstituted a minimal in vitro system with human components and revealed that NUB1 uses the intrinsic instability of FAT10 to trap its N-terminal ubiquitin-like domain in an unfolded state and deliver it to the 26S proteasome for engagement, allowing the degradation of FAT10-ylated substrates in a ubiquitin-independent and p97-independent manner. Using hydrogen–deuterium exchange, structural modeling and site-directed mutagenesis, we identified the formation of an intricate complex with FAT10 that activates NUB1 for docking to the 26S proteasome, and our cryo-EM studies visualized the highly dynamic NUB1 complex bound to the proteasomal Rpn1 subunit during FAT10 delivery and the early stages of ATP-dependent degradation. These findings identified a previously unknown mode of cofactor-mediated, ubiquitin-independent substrate delivery to the 26S proteasome that relies on trapping partially unfolded states for engagement by the proteasomal ATPase motor.

## Abstract (web summary)

Here, Arkinson et al. reconstitute NUB1-mediated FAT10 degradation by the human 26S proteasome and use biochemistry, cryo-EM and hydrogen–deuterium exchange to show that NUB1 acts as an ATP-independent chaperone to trap partially folded FAT10 for proteasome delivery.

## Main

The 26S proteasome degrades ubiquitinated substrate through a bipartite signal: a suitable ubiquitin modification, such as a polyubiquitin chain, and an unstructured initiation region of at least 20–25 residues that can enter the central channel of the proteasome for engagement by the AAA+ (ATP hydrolases associated with diverse cellular activities) motor^1–4^. The 26S proteasome comprises the 20S core particle, with sequestered proteolytic active sites, and the 19S regulatory particle, which includes ubiquitin receptor subunits, Rpn10, Rpn1 and Rpn13 (refs. ^5–8^), the deubiquitinase Rpn11 and the heterohexameric AAA+ motor that consists of the ATPase subunits Rpt1–Rpt6 (refs. ^9–11^). After substrate binding, the ATPase motor engages a substrate’s unstructured initiation region through conserved pore loops in its central channel and uses ATP hydrolysis-driven conformational changes for substrate unfolding and translocation into the core particle for cleavage^4^, while Rpn11 catalyzes the cotranslocational en bloc removal of ubiquitin modifications^12–14^. Substrates that lack unstructured initiation regions can be made accessible for proteasomal degradation through processing by the AAA+ protein unfoldase p97 (Cdc48 in yeast), which initiates unfolding and translocation on substrate-attached ubiquitin^15–17^.

Recently, several ubiquitin-independent pathways for proteasomal degradation have emerged, yet their detailed mechanisms remain unclear^18–20^. Here, we determine the mechanism for the ubiquitin-independent degradation of the ubiquitin-like modifier FAT10 (human leukocyte antigen-F-adjacent transcript 10). Predominantly expressed in immune cells, FAT10 regulates numerous cellular processes, including apoptosis and antigen presentation; in cancers, it promotes proliferation and metastasis formation^21–28^. FAT10 expression can be induced by virus infections or proinflammatory cytokines such as TNF and IFNγ (refs. ^21,23,29–32^). Unlike ubiquitin, FAT10 is not removed from substrates and recycled, but functions as both the targeting signal and a probable initiation region for degradation^30,33^. In its free and conjugated forms, FAT10 is rapidly degraded by the 26S proteasome, with an estimated half-life in cells of ~1 h (ref. ^30^). It contains two ubiquitin-like domains (UBLs) connected by a short linker and, while there is evidence for its degradation being ubiquitin independent^30,34,35^, other studies indicated ubiquitin-mediated targeting^36^. Interestingly, FAT10 does not contain any disordered segments long enough for proteasomal engagement and its degradation in vivo is not blocked by p97 inhibitors^33^, leaving the question of how it can bypass the requirement for a bipartite degradation signal.

The inflammation-induced protein NUB1 (NEDD8 ultimate buster 1) and its longer isoform NUB1L can accelerate FAT10 degradation^37,38^. NUB1 contains an N-terminal UBL domain and three C-terminal ubiquitin-associated domains (UBA1–UBA3), which may bind the 26S proteasome^35,39,40^. Whereas FAT10 is exclusively found in mammals, NUB1 variants are also present in flies and plants, suggesting a more conserved function that is potentially linked to the role of NUB1 in accelerating NEDD8 degradation^41^. The UBA domains of NUB1 appear critical for FAT10 binding, but they were claimed to be dispensable for facilitating FAT10 degradation, and it was suggested that a ternary complex with the 26S proteasome not involving direct FAT10–NUB1 interactions may be sufficient to drive FAT10 turnover^39^. The proteasomal ubiquitin receptors Rpn10 and Rpn1 were both postulated to bind the NUB1 UBL domain, and Rpn10 was also assumed to bind to the NUB1 UBA domains and the FAT10 UBL2 domain, suggesting some form of competing interactions or order of events that led to a confusing model for FAT10 recruitment^35,40,42^. Overall, the interaction of NUB1 with FAT10 and the 26S proteasome as well as the mechanisms of FAT10-mediated substrate degradation remained unclear.

Here, we reconstituted FAT10 degradation by the human *Homo sapiens* 26S (*hs*26S) proteasome and identified NUB1 as essential for its delivery and engagement by the ATPase motor. Using hydrogen–deuterium exchange with detection by mass spectrometry (HDX–MS), AlphaFold modeling, biochemical assays and cryo-electron microscopy (cryo-EM), we show that NUB1 functions as an ATP-independent chaperone that ‘traps’ partially unfolded FAT10 for insertion into the proteasomal processing channel. Furthermore, we revealed that FAT10 binding induces an open NUB1 conformation, in which the NUB1 UBL domain is undocked from the trap domain and, thus, available to specifically interact with the T2 site of the proteasome Rpn1 receptor subunit for FAT10 delivery. These data provide mechanistic insight into how a shuttle factor can mediate the turnover of its target substrates in a ubiquitin-independent manner and, more specifically, explain how NUB1 allows FAT10-modified proteins to bypass p97 requirements for engagement and degradation by the 26S proteasome.

## Results

### Proteasomal FAT10 degradation depends on NUB1

**Fig. 1:**
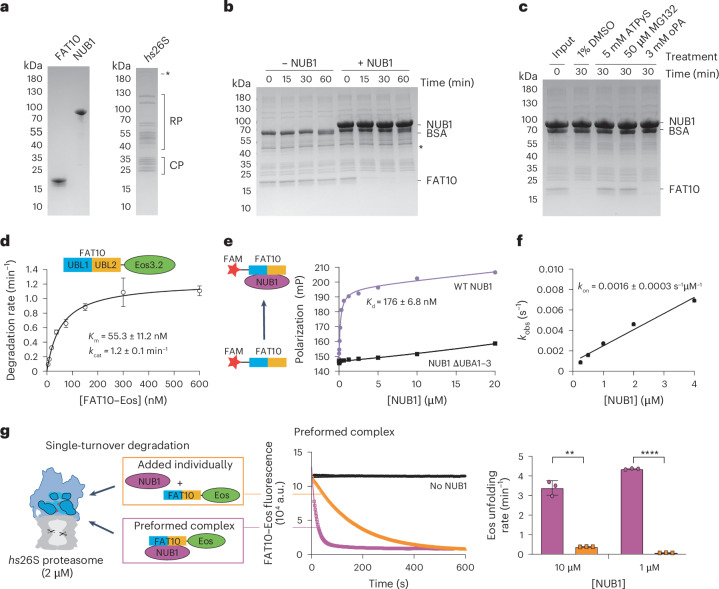
Ubiquitin-independent FAT10 degradation by the *hs*26S proteasome depends on NUB1 complex formation as the rate-limiting step in degradation. **a**, Coomassie-stained SDS–PAGE gels documenting the purity of recombinantly expressed human FAT10 (3 μg) and human NUB1 (1.5 μg) and endogenous *hs*26S proteasome purified from HEK293 cells (1.5 μg). CP, core particle; RP, regulatory particle. **b**, FAT10 degradation by the *hs*26S proteasome depends on the presence of NUB1. Coomassie-stained SDS–PAGE analysis of aliquots taken at different times during the degradation of FAT10 (5 μM) by the *hs*26S proteasome (100 nM) in the absence or presence of NUB1 (15 μM). **c**, Coomassie-stained SDS–PAGE analysis of FAT10 degradation by the *hs*26S proteasome in the presence of indicated concentrations of DMSO, the nonhydrolyzable ATP analog ATPγS, the proteasome inhibitor MG132 and the Rpn11 deubiquitinase inhibitor oPA. **d**, Michalis–Menten analysis of FAT10–Eos3.2 degradation (10–600 nM) by the *hs*26S proteasome (2 nM) in the presence of excess NUB1 (10 μM). Shown are the mean values and s.d. of the degradation velocity determined from the loss of Eos fluorescence for *n* = 3 technical replicates. **e**, Measurement of NUB1–FAT10 complex formation by fluorescence polarization. ^FAM^FAT10 (100 nM) was incubated with varying NUB1 concentrations for 45 min on ice before measuring the polarization. A truncated NUB1^ΔUBA1^^–^^3^ variant was used as a control for nonspecific binding. Data show the mean ± s.d. (*n* = 3 technical replicates). **f**, Kinetic analysis of the slow complex formation between FAT10 and NUB1. Shown are the apparent rate constants *k*_obs_ for the binding of ^FAM^FAT10 (20 nM) to NUB1 at varying concentrations (250–4,000 nM) as determined from the change in fluorescence polarization. Data show the mean ± s.d. (*n* = 3 technical replicates). **g**, Complex formation of NUB1 and FAT10 is the rate-limiting step in FAT10 degradation. Left, schematic for the experimental setups of the single-turnover degradation reactions, in which the *hs*26S proteasome (2 μM) was mixed with either the individual components (orange outline) of FAT10–Eos (100 nM) and excess NUB1 (1 or 10 μM) or preformed complexes (purple outline) of FAT10–Eos and NUB1 at the same concentrations. Middle, representative curves for the loss of FAT10–Eos fluorescence during the single-turnover degradation in the absence of NUB1 (black) or the presence of NUB1 (10 μM) without (orange) or with (purple) preincubation for 30 min. Right, mean values and s.d. for FAT10–Eos3.2 unfolding by the *hs*26S proteasome in the presence of NUB1 (1 or 10 μM) with (purple) or without (orange) preincubation for NUB1–FAT10 complex formation (*n* = 3 technical replicates). Statistical significance was calculated using an unpaired two-tailed Welch’s *t*-test. ****P < 0.000003 and **P = 0.005098. Source data

To determine how NUB1 mediates FAT10 degradation, we reconstituted this process in vitro with *Escherichia coli*-expressed full-length human FAT10 and NUB1, as well as the *hs*26S proteasome (Fig. 1a). Using SDS–PAGE, we monitored the degradation of FAT10 in the absence or presence of excess NUB1 and found that, at least in vitro, the rapid turnover of FAT10 strictly depends on NUB1 and does not require ubiquitination (Fig. 1b). Control experiments with the proteasome-specific inhibitor MG132, the slowly hydrolyzed ATP analog ATPγS or the Rpn11 inhibitor *ortho*-phenanthroline (oPA) confirmed that this degradation relies on proteolysis by the 20S core particle and ATP-dependent unfolding and translocation by the 19S regulatory particle, yet is independent of Rpn11 activity (Fig. 1c).

**Extended Data Fig. 1:**
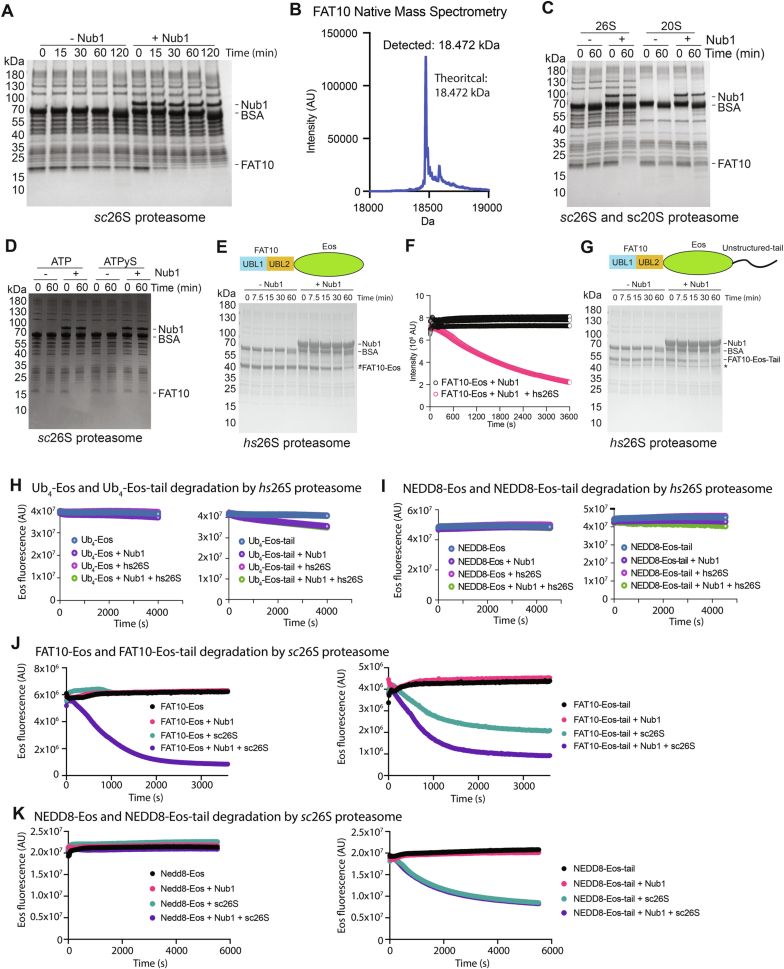
Supporting data for Fig. 1; NUB1 accelerates degradation of FAT10 for yeast proteasomes, does not accelerate degradation of Nedd8 fusions, and a disordered tail can bypass the requirement of NUB1 for degradation by yeast proteasomes but not human proteasomes. **a)** Coomassie-stained SDS-PAGE gel showing the time course for the degradation of FAT10 (10 μM) by *sc*26S proteasome (100 nM) with and without NUB1 (5 μM). **b)** Native mass spectrometry confirms the lack of truncations or modifications for *E. coli*-expressed human FAT10. **c)** Coomassie-stained SDS-PAGE gel showing end points of FAT10 degradation (10 μM) by *sc*26S proteasome (100 nM) or *sc*20S CP (100 nM) with or without NUB1 (5 μM). Minimal turnover by *sc*20S CP indicates that robust FAT10 degradation depends on ATPase-driven processing by 19S RP. **d)** Same assay as in C) in the presence of either ATP or ATPγS, with the latter confirming the dependence of FAT10 degradation on ATP-dependent processing. **e)** Degradation of FAT10-Eos (5 μM) and **g)** FAT10-Eos-tail (5 μM) by *hs*26S proteasome (100 nM) with and without NUB1 (15 μM) demonstrates that adding a disordered engagement sequence does not bypass the NUB1 requirement. **f)** Example traces for the FAT10-Eos (2.5 μM) degradation by hs26S proteasome (100 nM) in the presence of sub-stoichiometric NUB1 (0.5 μM) shows that NUB1 functions as a catalyst that itself is not degraded. **h)** Example traces showing the loss of Eos3.2 fluorescence during the degradation of Ub_4_-Eos (5 μM) and Ub_4_-Eos-tail (5 μM) by *hs*26S proteasome (100 nM) in the presence or absence of NUB1 (15 μM). While Ub_4_-Eos3.2-tail is robustly degraded with or without NUB1, Ub_4_-Eos is not. **i)** Same fluorescence-based assays as in G) except using NEDD8-Eos (left) and NEDD8-Eos-tail (right). Neither substrate is degraded by *hs*26S proteasome, even in the presence of NUB1. **j)** Left: Degradation of FAT10-Eos (2 μM) by *sc*26S proteasome (100 nM) in the absence or presence of NUB1 (5 μM) shows NUB1 dependence. Right: Degradation of FAT10-Eos-tail (2 μM) by *sc*26S proteasomes (100 nM) in the absence or presence of NUB1 (5 μM), indicating that a flexible tail on the substrate can bypass the NUB1 requirement for *sc*26S proteasomes. **k)** Same fluorescence-based assays as in I), except using NEDD8-Eos (left) and NEDD8-Eos-tail (right). NEDD8 binding to the yeast proteasome allows substrate delivery and degradation if there is a flexible initiation region for engagement. Source data

Interestingly, it was previously shown that overexpressed FAT10 is degraded by the 26S proteasome in yeast cells that naturally lack NUB1 and coexpressing NUB1 accelerated this in vivo degradation^35^. However, when reconstituting this process in vitro, we found that the 26S proteasome from yeast *Saccharomyces cerevisiae* (*sc*26S), similar to its human counterpart, cannot degrade FAT10 in an NUB1-independent manner (Extended Data Fig. 1a). Hence, other mechanisms may aid FAT10 degradation in yeast cells, for instance, ubiquitination and/or Cdc48-mediated unfolding. FAT10 was previously reported to easily aggregate^33,43^ and be susceptible to degradation by the isolated 20S core particle after longer incubations in vitro^44^. However, our *E.* *coli*-expressed wild-type FAT10 was highly soluble, well behaved and not truncated during expression or purification (Extended Data Fig. 1b). It was only very slowly degraded by the yeast 20S core particle alone (Extended Data Fig. 1c) or by the *sc*26S proteasome in the presence of ATPγS (Extended Data Fig. 1d), likely because of its intrinsic lability and some extent of spontaneous unfolding over the 60-min period of the experiment. These observations support the validity of our findings that FAT10 is rapidly and specifically degraded by the *hs*26S and *sc*26S proteasomes in a process that strongly depends on ATP and NUB1.

### NUB1 targets FAT10 conjugates for degradation

To measure the kinetics of FAT10 turnover by the *hs*26S proteasome, we designed a reporter construct with FAT10 fused to the N terminus of mEos3.2 (FAT10–Eos), which allowed monitoring the unfolding and degradation through the loss of Eos fluorescence. mEos3.2 lacks unstructured initiation regions suited for proteasomal engagement, such that it is not degraded without prior Cdc48-mediated unfolding even in its ubiquitinated form^45^. However, FAT10–Eos was robustly degraded by the *hs*26S proteasome in the presence of NUB1 (Extended Data Fig. 1e). As expected for a fusion with the hard-to-unfold Eos domain, the observed rate was lower than for the isolated FAT10 (Fig. 1b and Extended Data Fig. 1e). Michaelis–Menten analysis of FAT10–Eos degradation in the presence of saturating NUB1 concentrations revealed a Michaelis constant (*K*_m_) of 55.3 ± 11.2 nM and a turnover number (*k*_cat_) of 1.2 ± 0.1 min^−1^ (Fig. 1d), in alignment with previously reported velocities for the degradation of ubiquitinated mEos3.2 with an unstructured tail by *sc*26S proteasomes^4^. Importantly, NUB1 acts as a true cofactor for FAT10 substrate degradation, as it is not turned over (Fig. 1b) and functions in substoichiometric amounts (Extended Data Fig. 1f).

We next tested whether FAT10 could act as an autonomous targeting signal and whether the addition of a long disordered tail on FAT10–Eos may bypass the NUB1 requirements for proteasomal engagement. However, FAT10–Eos–tail remained undegraded in the absence of NUB1 (Extended Data Fig. 1g), indicating that FAT10 alone is insufficient for either recruitment or initiation. Similarly, fusing the NUB1 UBL domain to the N terminus of Eos–tail (UBL–Eos–tail) did not allow its degradation (Supplementary Fig. 1). To explore this further, we created a linear fusion of four ubiquitin moieties with Eos–tail (Ub_4_–Eos–tail), which, in contrast to the untailed Ub_4_–Eos control, was degraded by the *hs*26S proteasome, albeit slowly (Extended Data Fig. 1h). These results indicate that, without NUB1, FAT10 fails to efficiently interact with the *hs*26S proteasome or present the C-terminal tail on Eos for proteasomal engagement. NUB1 was also shown to accelerate NEDD8 degradation, but neither NEDD8–Eos nor NEDD8–Eos–tail showed turnover in the absence or presence of NUB1 (Extended Data Fig. 1i), suggesting that a specific FAT10–NUB1 interaction drives the degradation of FAT10-ylated proteins by the *hs*26S proteasome.

Interestingly, FAT10–Eos–tail and NEDD8–Eos–tail were degraded by the *sc*26S proteasome in the absence of NUB1 (Extended Data Fig. 1j,k), indicating that both modifiers are sufficient for turnover by the yeast enzyme, provided that a long disordered tail is present on the substrate. This suggests that yeast proteasomes are more promiscuous in UBL domain binding; indeed, previous work showed that many types of UBL-fused substrates can be degraded by the *sc*26S proteasome^46–49^. Importantly, FAT10 is not sufficient to mediate the degradation of tailless FAT10–Eos by the *sc*26S proteasomes, which still depends on NUB1 (Extended Data Fig. 1j). For degradation of FAT10-ylated substrates by the *hs*26S proteasome, it can be concluded that NUB1 is required for both the specific recruitment and the initiation.

### FAT10 and NUB1 slowly form a high-affinity complex

On the basis of our size-exclusion chromatography (SEC) experiments, FAT10 and NUB1 form a stable 1:1 complex (Supplementary Fig. 2a). To determine their affinity, we N-terminally labeled FAT10 with fluorescein amidite (FAM) and used fluorescence polarization measurements. When mixing this ^FAM^FAT10 with excess NUB1, we detected a slow increase in polarization that was dependent on NUB1 concentration (Fig. 1e,f and Supplementary Fig. 2b) and titrating NUB1 revealed a dissociation constant (*K*_d_) of 176.0 ± 6.8 nM for the NUB1–^FAM^FAT10 complex (Fig. 1e). This represents an approximate dissociation constant, as some aggregation occurred at higher NUB1 concentrations, potentially caused by the nature of the NUB1–FAT10 interaction or the hydrophobic FAM label on FAT10. Deleting the UBA domains of NUB1 eliminated FAT10 binding (Fig. 1e), which agrees with previous reports^39^ and confirms a specific NUB1–FAT10 interaction. Measuring the kinetics revealed an association constant of *k*_on_ = 0.0016 ± 0.003 s^−1^ μM^−1^ (Fig. 1f).

To further explore the importance of this slow complex formation, we conducted single-turnover FAT10–Eos degradation assays with excess *hs*26S proteasome, which provided insight into the processes before Eos unfolding. Mixing FAT10–Eos (100 nM) with saturating amounts of NUB1 (10 µM) and *hs*26 proteasome (2 µM; Fig. 1d), we observed a single-exponential decay of Eos fluorescence with a time constant of *τ* = 162 ± 4.3 s, equivalent to a degradation rate of *k*_unfold_ = 0.37 min^−1^ (Fig. 1g). This represents the time required for complex formation, binding to the proteasome and initial unfolding of the Eos β-barrel. By contrast, when we performed the experiment at identical concentrations but preincubated FAT10–Eos and NUB1 for 20 min before *hs*26 proteasome addition, we detected fast processing with a time constant of *τ*_fast_ ≈ 18.0 ± 2.2 s, equivalent to a rate of *k*_unfold_ = 3.3 min^−1^. We also observed a low-amplitude (9%) second phase with a time constant of *τ*_slow_ = 192 ± 31.7 s (Fig. 1g), likely because of a minor population of aggregated complexes. Importantly, the dominant first phase of the degradation reaction proceeded almost an order of magnitude faster than the unfolding observed without preincubating NUB1 and FAT10–Eos (*k*_unfold_^(preincubation)^ = 3.3 min^−1^ versus *k*_unfold_ = 0.37 min^−1^; Fig. 1g). We validated this by reducing the NUB1 concentration to 1 µM, which led to even slower degradation kinetics when not preincubating NUB1 and FAT10–Eos (Fig. 1g), as their complex formation is rate determining and concentration dependent. By contrast, degradation still progressed rapidly when using the NUB1–FAT10–Eos complex that was preformed by incubating the identical concentrations of 100 nM FAT10 and 1 µM NUB1 before addition of the proteasome (Fig. 1g). Together, the polarization-based binding measurements and the degradation studies under single-turnover conditions demonstrated that the complex formation between NUB1 and FAT10 is slow and rate determining for proteasomal turnover.

### NUB1 traps partially unfolded FAT10 by binding a β-strand

**Fig. 2:**
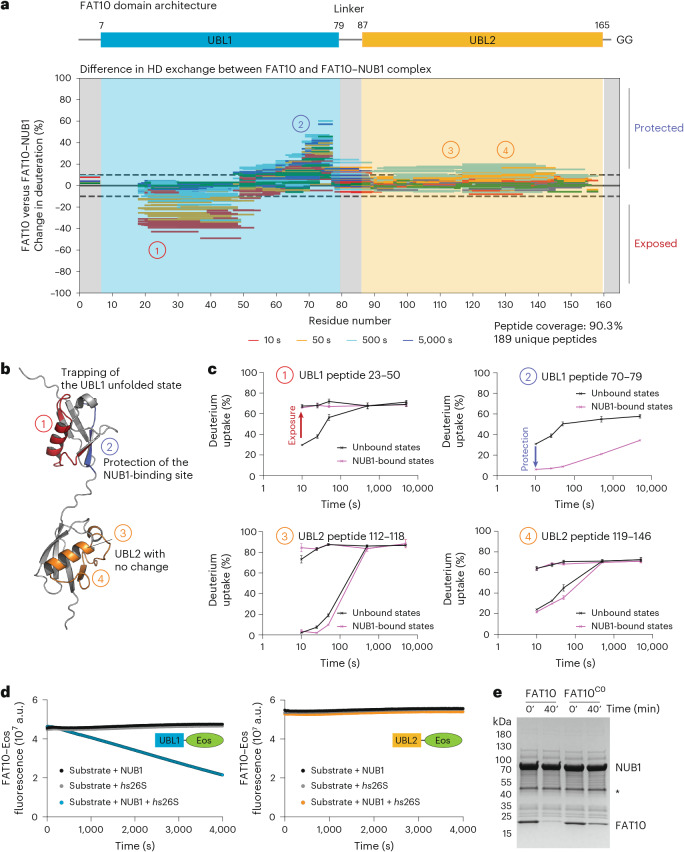
NUB1 stabilizes the unfolded state of the FAT10 UBL1. **a**, Top, schematic of the FAT10 domain architecture, with the UBL1 domain shown in cyan and the UBL2 domain in orange. Bottom, Wood’s plot representation of the percentage changes in deuteration between free FAT10 and the FAT10–NUB1 complex, with decreased uptake (protection) shown above 0% and increased uptake (exposure) shown below 0%. For bimodal peptides, only less exchanged peaks are shown for comparison. The time of HDX is indicated by different colors. Changes in deuteration between the dotted lines were considered to be not significant. Encircled numbers indicate four representative peptides in the UBL1 and UBL2 domains that become exposed (red), become protected (blue) or show no change upon FAT10–NUB1 complex formation. Their positions within the FAT10 structural model are shown in **b** and their deuterium uptake kinetics are depicted in **c**. **b**, Structure representation of FAT10, indicating the positions of peptide 1 (red) that shows increased exposure upon complex formation with NUB1, peptide 2 (blue) that represents the last β-strand in the UBL1 domain and gets protected by NUB1 binding and peptides 3 and 4 (orange) in the UBL2 domain that are unaffected by NUB1 binding. **c**, Deuterium uptake plots for peptides 1–4 depicted in **a**,**b**. Shown are the means and s.d. for the percentages of deuterium uptakes based on the theoretical maximum deuteration for each peptide, determined from *n* = 3 technical replicates. Top left, peptide 1 shows a bimodal distribution of deuterium uptake in the absence of NUB1 and becomes unimodally exposed throughout the time course upon NUB1 binding. Top right, peptide 2 shows unimodal exchange and becomes protected upon NUB1 binding. Bottom left and right, the bimodal uptake behavior for peptides 3 and 4 throughout the time course remains unchanged after FAT10 binding NUB1. **d**, Example Eos fluorescence traces for degradation of FAT10^ΔUBL2^–Eos (left) and FAT10^ΔUBL1^–Eos (right) by the *hs*26S proteasome with (blue and orange) or without (gray) excess of NUB1 (15 μM). **e**, Coomassie-stained SDS–PAGE gel showing the endpoints for the multiple-turnover degradation of wild-type FAT10 and the thermodynamically stabilized cysteine-free FAT10^C0^ by the *hs*26S proteasome after preincubation with excess NUB1 (15 μM). Source data

**Extended Data Fig. 2:**
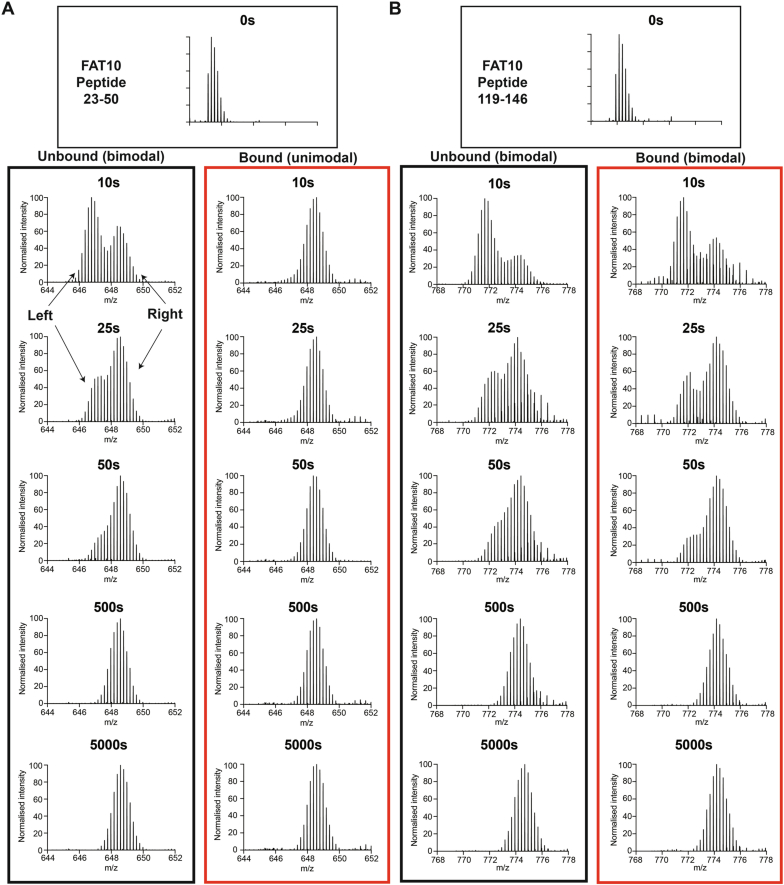
Example mass spectra from HDX-MS experiments of FAT10 versus FAT10 in the presence of excess NUB1. Intensity-normalized mass spectra for example peptides in FAT10’s UBL1 (**a**) and UBL2 domain (**b**) show bimodal deuterium uptake with two peaks, a left and a right distribution. The right distribution is fully exchanged, whereas the left peak shifts to the right and, in addition, its ratio relative to the right peak lowers over time. This behavior indicates that the peptide exchanges with a combination of EX1 kinetics (leading to a change in the peak ratio) and EX2 kinetics (leading to an average peak movement from left to right). The bimodal nature suggests that there are multiple species of FAT10, folded and unfolded, which transition on the order of minutes based on the EX1 kinetics of FAT10. In the presence of excess NUB1, the UBL1 peptide (A) shows unimodal behavior with the peptide signal shifted to the right, indicating a fully unfolded and fast exchanging species. In contrast, the UBL2 peptide (B) remains largely unaffected in its bimodal behavior by NUB1. Source data

The 26S proteasome requires an unstructured initiation region for substrate degradation, which prompted us to investigate whether NUB1 binding provides FAT10 with a flexible segment. We analyzed changes in conformation and solvent accessibility of FAT10 upon NUB1 binding using HDX–MS. Protonated FAT10 was incubated in D_2_O with or without excess NUB1, followed by quenching of the exchange, pepsin digestion and liquid chromatography (LC)–MS peptide detection, in which we achieved excellent peptide coverage across the FAT10 sequence (Fig. 2a and Supplementary Table 1). Several peptides from both UBL domains of FAT10 exhibited bimodal distributions in the absence of NUB1, indicating coexisting exposed (unfolded) and protected (folded) states, consistent with the previously observed dynamic nature of FAT10 (refs. ^33,43^) (Fig. 2b,c and Extended Data Fig. 2). The HDX of these peptides showed a mixture of EX1 and EX2 kinetics (EX1: exchange mechanism 1, with the intrinsic rate of exchange faster than local refolding; EX2: exchange mechanism 2, with the intrinsic rate of exchange slower than local refolding), which made it difficult to determine the relative populations for each state (Extended Data Fig. 2a). We, therefore, used the left peak from bimodal distributions for all peptides to compare the differences between free FAT10 and the FAT10–NUB1 complex (Fig. 2a). Notably, NUB1 binding caused an exposure of peptides throughout the FAT10 UBL1 domain, except for the last β-strand, which showed protection in the presence of NUB1. This suggests that NUB1 induces or traps an unfolded state of UBL1. We focused on the presence or absence of bimodal distributions to describe the effect of NUB1 binding on FAT10 and selected four example peptides, two from each UBL domain. Three of the peptides displayed clear bimodal deuterium uptake in the absence of NUB1, with a slowly exchanging and a fully exchanged population throughout all early time points, whereas the fourth peptide, derived from the last β-strand of UBL1, showed primarily unimodal distribution (Fig. 2b,c). Remarkably, NUB1 binding eliminated the bimodal distribution for the first UBL1 peptide, leaving only the fully exposed population, whereas both UBL2 peptides stayed unaffected and retained bimodal exchange (Fig. 2b,c and Extended Data Fig. 2a,b). This indicates that NUB1 specifically interacts with UBL1 and has no effect on UBL2, which would be consistent with previous studies indicating that the UBL1 and UBL2 domains of FAT10 represent independently folding domains with no considerable interactions^33^. Interestingly, the slowly exchanging UBL1 population in the absence of NUB1 showed deuterium uptake kinetics in the minute range, similar to the time constants we observed for the NUB1–FAT10 complex formation in our fluorescence polarization and single-turnover degradation experiments (Fig. 1f,g). We, therefore, propose that NUB1 uses conformational selection to bind and trap the spontaneously unfolding UBL1 domain of FAT10 rather than actively inducing unfolding. The last β-strand of UBL1, which shows protection from deuterium exchange upon NUB1 binding, follows the conserved H75 residue; therefore, we term it H75^β-strand^ (Fig. 2b,c). This single β-strand appears to be the NUB1 binding site within FAT10.

### NUB1 delivers the FAT10 UBL1 domain for proteasomal engagement

Because the FAT10 UBL1 domain seems to provide both the binding site for NUB1 and the disordered initiation region for engagement by the proteasome, we tested whether the presence of this domain is sufficient to facilitate the degradation of mEos3.2 in the presence of NUB1. Indeed, our single-turnover experiments showed that FAT10^ΔUBL2^–Eos is degraded in an NUB1-dependent manner, albeit ~2-fold more slowly than full-length FAT10 ($\tau^{{\rm{FAT}10}^{\Delta{\rm{UBL}}2}}$ = 54.4 ± 4.5 s versus *τ*^FAT10-WT^ = 28.6 ± 0.9 s; Supplementary Fig. 3a). These findings indicate a kinetic effect of the FAT10 UBL2 domain on the rate-limiting step during initiation rather than a contribution to the binding affinity. UBL2 may form minor interactions that help orient the NUB1–FAT10 complex for initiation or act as a spacer between the NUB1-bound UBL1 domain and the protein substrate to prevent steric clashes with the proteasome. By contrast, FAT10^ΔUBL1^–Eos showed no degradation (Fig. 2d), even after attaching an unstructured segment to the C terminus (FAT10^ΔUBL1^–Eos–tail; Supplementary Fig. 3b). It was previously proposed that the degradation of FAT10 is initiated at its N terminus^33^ and, to prove this in our reconstituted system, we blocked the N terminus with a fusion to Smt3, the yeast homolog of the small ubiquitin-like modifier, SUMO. No degradation was observed for this Smt3–FAT10 construct (Supplementary Fig. 3c), confirming that a free N terminus is critical for FAT10 degradation. On the basis of our HDX–MS experiments and the slow NUB1–FAT10 complex formation, we hypothesized that the intrinsic lability and spontaneous unfolding of the FAT10 UBL1 domain are crucial for NUB1 binding and consequently degradation by the proteasome. Previous studies showed that a quintuple cysteine-less mutant, FAT10^C0^, with increased thermodynamic stability exhibited decreased degradation in vivo^33^. We, therefore, generated FAT10^C0^ and, indeed, observed that degradation by the *hs*26S proteasome was strongly decelerated (Fig. 2e) and NUB1 binding was undetectable by fluorescence polarization (Supplementary Fig. 3d). Together, these results demonstrate that the FAT10 UBL1 domain functions as a degradation initiation region whose structural instability allows NUB1 to bind and trap an unfolded state for engagement by the 26S proteasome.

### NUB1 domains form an expandable channel for FAT10 binding

**Fig. 3:**
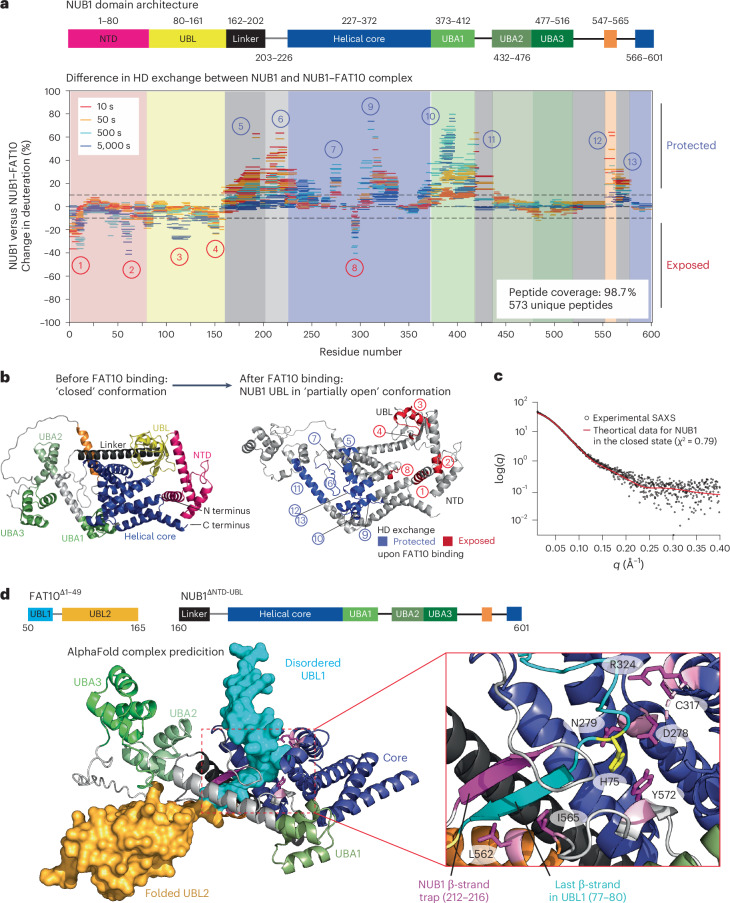
HDX and SAXS reveal conformational changes in NUB1 upon trapping FAT10 in a partially unfolded state. **a**, Top, schematic of the NUB1 domain architecture with residue numbers indicated. Bottom, Wood’s plot representation of the percent changes in deuteration between free NUB1 and the NUB1–FAT10 complex, with decreased deuterium uptake (protection) upon FAT10 complex formation for individual peptides shown above 0% and increased uptake (exposure) shown below 0%. The time of HDX is indicated by different colors. Changes between the dotted lines were considered to be not significant. Encircled numbers indicate specific peptides that get exposed (red) or protected (blue) upon FAT10 complex formation and their position within the NUB1 structural model is shown in **c**. **b**, ColabFold-generated AlphaFold models of NUB1 show the domain architecture and distinct conformational states. Assigned domains and their boundaries based on AlphaFold models are indicated by colors and consistent with the domain predictions shown in **a**. The positions of the NTD and UBA2, UBA3 and UBL domains vary between different models, in which the UBL domain is observed docked against the core domain in a closed NUB1 conformation (left) or more exposed in a partially open NUB1 conformation (right). Right, selected peptides highlight key changes in the partially open state of NUB1 upon FAT10 binding, with more protected peptides shown in blue and more exposed peptides in red mapped onto the NUB1 structure. Peptides with increased protection in the FAT10-bound complex are generally localized within areas and linkers that line a channel formed between the helical core domain and the UBA domains of NUB1. **c**, SAXS profile for isolated full-length NUB1. Shown as the red line is the theoretical scattering curve for the NUB1 closed state as predicted by AlphaFold and shown in **c**, with the UBL domain docked against the core. **d**, AlphaFold-Multimer structure prediction of the NUB1–FAT10 complex. Top, schematics for the N-terminally truncated variants of NUB1 and FAT10 used for this structure prediction. Bottom left, structural model for the NUB1–FAT10 complex, with NUB1 shown in ribbon representation, colored as in the schematic at the top, and FAT10 depicted in surface representation, with the UBL1 domain threaded through NUB1 and trapped in an unfolded state. Bottom right, zoomed-in view of the NUB1–FAT10 interaction, where the last β-strand of the FAT10 UBL1 domain (cyan ribbon) is trapped by forming an antiparallel β-sheet with the β-strand linker (purple ribbon) of NUB1. FAT10 H75 (yellow stick representation) is coordinated by D278, N279 and Y572 of NUB1 and additional NUB1 residues relevant for the interaction with FAT10 are shown in purple stick representation. Source data

**Extended Data Fig. 3:**
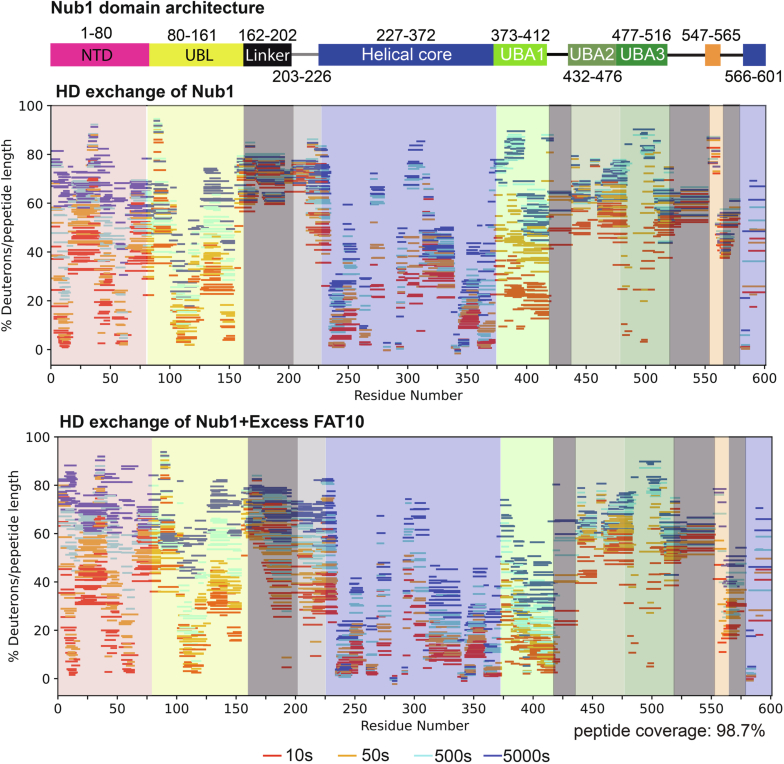
Woods plots for the deuteration levels of NUB1 in the absence or presence of excess FAT10. Displayed are the deuteration levels in percent for each peptide found in both NUB1 alone and NUB1 with excess FAT10 at time point of 10–5000 s after mixing with D_2_O. Each peptide is positioned in alignment with the amino acid sequence of NUB1 on the X-axis and the domain schematic above. The y-axis shows deuteration levels in percent relative to the theoretical minimum and maximum amide-proton exchange for each peptide. Source data

**Extended Data Fig. 4:**
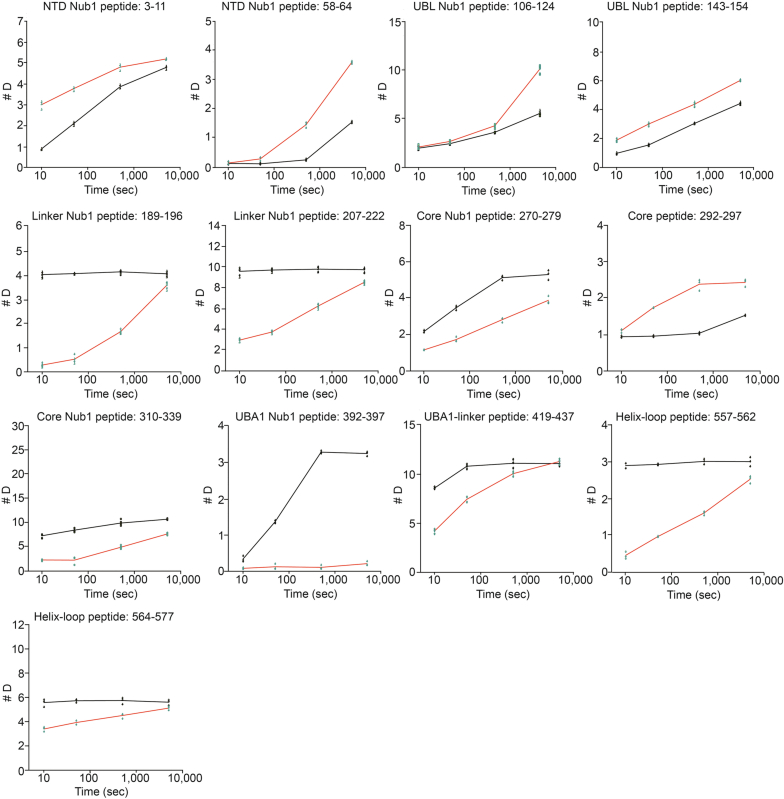
Deuterium uptake plots for selected NUB1 peptides. Plots show the deuterium uptake at 10, 50, 500 and 5000 s for individual peptides of NUB1 (black curves) compared to NUB1 in the presence of FAT10 (red curves) for n = 3 technical replicates. Source data

On the basis of annotated domains^39^ and secondary-structure predictions^50^, NUB1 contains an N-terminal domain (NTD) followed by an UBL domain attached through helical and unstructured linkers to a core domain. This core domain leads into the UBA1 domain, which is connected through another helical linker to the UBA2 and UBA3 domains, followed by a disordered region and two C-terminal helices. To assess changes in the conformation and solvent accessibility of NUB1 upon FAT10 binding, we performed HDX–MS experiments with protonated NUB1 in the absence or presence of excess FAT10. FAT10 binding caused changes in HDX across the entire sequence of NUB1, with the differences at various time points shown in Fig. 3a (Supplementary Table 1). Slow-exchanging peptides for unbound and FAT10-bound NUB1 correlated well with folded domains, while fast-exchanging peptides matched predicted linker regions (Fig. 3a and Extended Data Fig. 3). The observed differences in HDX profiles indicate considerable conformational changes, including an exposure of peptides in the NUB1 NTD and UBL domains. Deuterium uptake plots for selected NUB1 peptides in the absence and presence of FAT10 are shown in Extended Data Fig. 4.

**Extended Data Fig. 5:**
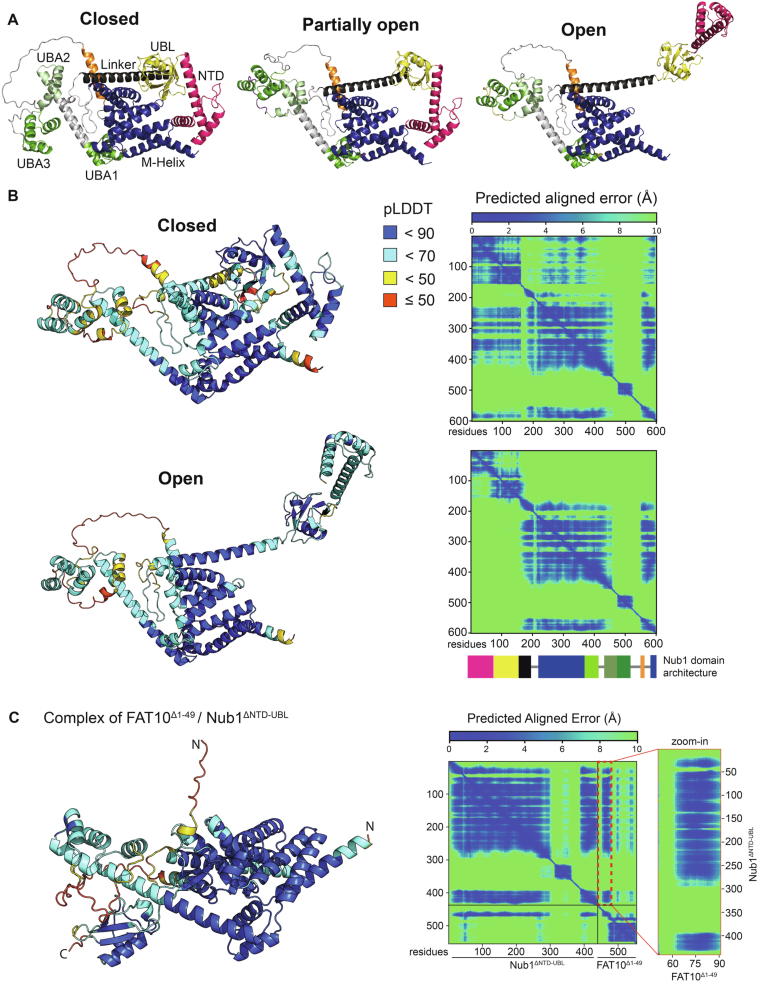
AlphaFold scores for selected NUB1 model predictions. **a)** The google notebook of Collabfold^64^, which is based on the AlphaFold algorithm, was used to generate 5 models of NUB1. Models with notable differences were selected and named ‘open’, ‘partially open’, and ‘closed’ based on the position of NUB1’s ubiquitin-like (UBL) domain. Models are colored as in Fig. 3b, with the N-terminal domain (NTD) in pink, the UBL domain in yellow, the helical linker in black, the disordered loop in grey (referred to as the beta-strand linker or bs-Trap), the core body in blue, and the three Ubiquitin-associated (UBA) domains in shades of green. **b)** AlphaFold models for the ‘closed’ state (top left) and ‘open’ state (bottom left) of NUB1 are colored based on predicted Local Distance Difference Test (pLDDT) scores. In brief, scores above 90 are considered as very accurate for local features and potentially side chains, 70–90 as good for overall fold with secondary structure, 50-70 as low confidence including the visual representation, and below 50 as likely disordered until in the right context. Right: Predicted aligned errors up to 10 Å for the closed and open states of NUB1, which describe the confidence in the relative positions of two residues and in domain-to-domain packing within the predicted structures. **c)** Left: AlphaFold-multimer model for the NUB1/FAT10 complex colored based on pLDDT scores. Right: predicted aligned error (PAE) depicts the quality of the AlphaFold-multimer predictions for domain docking and protein-protein interactions. The zoomed-in section for residues 50-92 of FAT10^Δ1-49^ highlights the confidence in the interaction between NUB1 and the last beta strand in FAT10’s UBL1 domain.

AlphaFold structure predictions identified at least three conformations for isolated NUB1 (‘closed’, ‘open’ and ‘partially open’) based on UBL domain positioning (Fig. 3b and Extended Data Fig. 5a,b). The core domain, UBA domains and their linkers form a loop structure that is anchored by interactions between the C-terminal helices and the core. This explains why truncated NUB1 lacking the C-terminal helices exhibited low solubility, while variants with deleted NTD-UBL (NUB1^ΔNTD-^^UBL^ and NUB1^ΔNTD-^^UBL^^–^^linker^) or deleted UBA domains (NUB1^ΔUBA1^^–^^3^) remained stable and soluble. Thus, NUB1 has three segments: the NTD-UBL, the core domain and the looped-out region containing UBA1, UBA2 and UBA3. A flexible linker between UBA3 and the C-terminal helices appears to enable the looped region to open and close.

Using SEC–SAXS (small-angle X-ray scattering), we determined the best conformational representation for the unbound NUB1 and validated the extent of NTD-UBL exposure. SAXS analysis showed the best fit for the closed UBL conformation, while the partially open state gave a reasonable yet not ideal fit and the fully open state fitted poorly (Fig. 3c and Supplementary Fig. 4). Hence, unbound NUB1 predominantly adopts the closed conformation, with its UBL domain bound to the core. Slight deviations in the closed-state fit likely originate from an ensemble of conformations or flexibility in regions such as UBA2–UBA3, which exhibit lower confidence scores in the AlphaFold model and may allow for an expandable channel. Notably, the protection observed in our HDX–MS experiments suggests that FAT10 interacts with regions lining this channel between the NUB1 core body and UBA domains, including a conserved flexible linker (Fig. 3a,b and Supplementary Fig. 5). Furthermore, FAT10 binding increases exposure of the NUB1 UBL domain and a region on the NUB1 core that the UBL domain contacts in the closed conformation. FAT10 appears to trigger the exposure of an interface on the NUB1 UBL domain that is equivalent to the I44 patch of ubiquitin, an area typically involved in ubiquitin receptor binding. Our results, thus, indicate that FAT10 binding induces a more open conformation in which the NUB1 UBL domain can interact with a proteasomal receptor.

### Structural model for NUB1 with trapped FAT10

To further explore the NUB1–FAT10 complex and corroborate HDX–MS-based models, we used AlphaFold-Multimer^51^ for structure predictions of truncated FAT10 (residues 50–166) and NUB1 (residues 161–600) (Fig. 3d and Extended Data Fig. 5c) and their full-length versions (Supplementary Fig. 6). Several confident models showed that FAT10 H75^β-strand^ inserted into a channel between the NUB1 helical core and UBA domains, while other parts of the FAT10 UBL1 domain remained disordered (Fig. 3d). These predicted interactions are consistent with our HDX–MS results (Figs. 2a and 3a), which showed HDX protection in multiple NUB1 regions surrounding the FAT10 H75^β-strand^. The H75^β-strand^ forms an antiparallel β-sheet with NUB1 residues 212–216, part of the unstructured linker (residues 211–223) between the UBL and core domains that exhibited strong HDX protection upon FAT10 binding; we refer to this as the β-strand trap (bs-trap) (Figs. 2c and 3d).

**Fig. 4:**
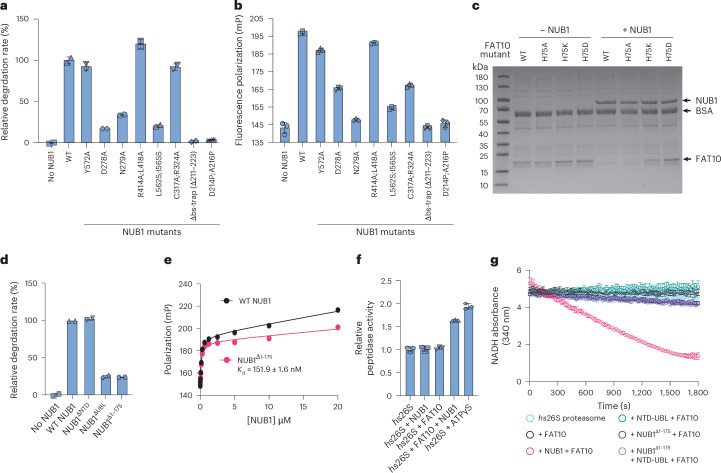
NUB1 uses a bs-trap to bind FAT10 and its UBL domain for interaction with the proteasome. **a**, Relative rates for the multiple-turnover degradation of FAT10–Eos (1 μM) by the *hs*26S proteasome (100 nM) in the presence of wild-type NUB1 (set to 100%) or NUB1 mutants (5 μM). Shown are the mean values and s.d. for *n* = 3 technical replicates. **b**, Fluorescence polarization as a readout for the binding of ^FAM^FAT10 (100 nM) to wild-type NUB1 and its mutants (5 μM) after preincubation for 30 min. **c**, Coomassie-stained SDS–PAGE gel showing the endpoints for the degradation of FAT10 wild type and its H75 mutants (10 μM) by the *hs*26S proteasome (100 nM) in the absence and presence of NUB1 (2.5 μM) after 30 min. **d**, Relative rate of FAT10–Eos degradation (1 μM) by the *hs*26S proteasome (100 nM) in the presence of various NUB1 truncation mutants (5 μM). Bar graphs show the mean values and error bars represent the s.d. for *n* = 3 technical replicates, normalized to the degradation in the presence of wild-type NUB1. **e**, Fluorescence polarization measurement of the NUB1–FAT10 complex formation indicates that the NUB1 UBL domain is dispensable for FAT10 binding. ^FAM^FAT10 (100 nM) was incubated with varying concentrations of wild-type NUB1 or NUB1^Δ1^^–^^175^ for 45 min on ice before measuring the polarization. Data show the mean ± s.d. (*n* = 3). **f**, Peptidase activity of the *hs*26S proteasome in the presence of FAT10, NUB1, the FAT10–NUB1 complex or ATPγS instead of ATP. Shown are the mean values and s.d. of the peptidase activities for *n* = 3 technical replicates, normalized to the activity of *hs*26S proteasome in the presence of ATP. **g**, Example traces for the depletion of NADH in a coupled ATP hydrolysis assay with the *hs*26S proteasome in the absence or presence of FAT10, NUB1, NUB1^Δ1^^–^^175^ or an NTD-UBL fragment of NUB1. Source data

In addition to backbone interactions between these β-strands, H75 of FAT10 is coordinated by conserved NUB1 residues, D278, N279 and Y572 (Fig. 3d), which are also protected from HDX in the presence of FAT10. As most contacts between H75^β-strand^ and bs-trap are mediated by backbones, we disrupted them by either deleting the bs-trap or substituting two residues with proline. Both NUB1^Δbs-trap^ (Δ211–223) and NUB1^D214P;A216P^ abolished degradation of FAT10–Eos (Fig. 4a) and showed no binding to ^FAM^FAT10 (Fig. 4b). Alanine substitutions of several NUB1 residues predicted by AlphaFold-Multimer to coordinate FAT10 H75^β-strand^ also compromised FAT10–Eos degradation and ^FAM^FAT10 binding (Fig. 4a,b and Supplementary Fig. 7a,b).

In addition to NUB1 regions that directly contact FAT10, we observed HDX protection upon FAT10 binding for a helix in the UBA1 domain that is positioned right behind Y572 (Supplementary Fig. 6c). The UBA1 domain likely stabilizes the region around Y572 that coordinates FAT10 H75, whereas, in the absence of FAT10, this domain appears more mobile, potentially facilitating FAT10 insertion into the NUB1 channel. Consistently, deletion of the NUB1 UBA1 domain inhibited FAT10 degradation, whereas deleting UBA2 and UBA3 had no major effects (Supplementary Fig. 6d). Lastly, substitutions of FAT10 critical H75 residue (H75A, H75D and H75K) all caused compromised NUB1 binding (Supplementary Fig. 3d), with H75D and H75K abolishing degradation (Fig. 4c). The H75A substitution had weaker inhibitory effects on turnover than expected from its NUB1-binding defects (Fig. 4c and Supplementary Fig. 3d), possibly because of additional interactions in the ternary complex with the proteasome that allow substrate delivery and degradation despite a more dynamic FAT10–NUB1 binding.

SAXS experiments helped further distinguish between AlphaFold-Multimer models of the full-length NUB1–FAT10 complex. While the fully open state poorly fit the SAXS data, a complex model with a partially open NUB1 UBL domain fit much better (Supplementary Fig. 8). However, AlphaFold models do not consider the flexibility and disorder of the FAT10 UBL domains when bound to NUB1 or the possibility of multiple NUB1 UBL conformations once released from the core and our SAXS data likely represent an ensemble of NUB1–FAT10 states. Interestingly, AlphaFold-Multimer models of FAT10-bound NUB1^ΔUBL^ gave an excellent fit to the corresponding experimental SAXS data (Supplementary Fig. 9), indicating that, in full-length NUB1, the UBL domain adopts multiple conformations.

Overall, the SAXS experiments effectively supported the AlphaFold-Multimer models for the NUB1–FAT10 complex, which are also in excellent agreement with our mutagenesis and HDX–MS results. This provides a structural picture of FAT10 bound by NUB1, which acts as an ATP-independent chaperone to trap the last β-strand in the FAT10 UBL1 domain and stabilize the unfolded state for proteasomal engagement and degradation.

### The NUB1 UBL domain allows FAT10 delivery to the proteasome

The results presented above indicate that FAT10 binding induces a partially open state of NUB1, allowing its UBL domain to contact a proteasomal receptor. In this model, uncomplexed NUB1 is in a closed state and not competing with FAT10-bound NUB1 for proteasome binding. To test this, we made NUB1 mutants lacking the NTD and/or the UBL domain (NUB1^ΔNTD^, NUB1^ΔUBL^ and NUB1^Δ1^^–^^175^) and analyzed their activity in facilitating FAT10–Eos degradation. Whereas the NTD deletion had no effect, eliminating the UBL domain strongly compromised FAT10 degradation (Fig. 4d and Supplementary Fig. 10a). As expected, deleting the NTD and UBL domain in NUB1^Δ1^^–^^175^ did not affect its binding of ^FAM^FAT10 (Fig. 4e), demonstrating that the NUB1 UBL domain is dispensable for FAT10 binding but critical for robust FAT10 degradation by the 26S proteasome. Consistent with NUB1 residing in a closed conformation with its UBL domain tucked in and unable to contact the proteasome, we did not observe inhibition of NUB1-mediated FAT10 degradation when higher concentrations of free NUB1 were present (Fig. 1b,d,g).

UBL domains, including those of NUB1, were previously reported to allosterically activate proteasomal peptidase or ATPase activities^40,52^. However, we observed a peptidase activity stimulation of the *hs*26S proteasome only when FAT10 and NUB1 were added together, whereas they had no effect individually (Fig. 4f). This suggests that stimulation requires either a FAT10-bound, partially open conformation of NUB1 with an exposed UBL domain or substrate engagement and degradation, which shifts the proteasome from nonprocessing to processing states with an open 20S peptidase gate. To investigate this further, we measured proteasomal ATPase activity in the absence and presence of FAT10 and/or NUB1 (Fig. 4g and Supplementary Fig. 10b). The *hs*26S proteasome alone had undetectable ATPase activity, which may be a consequence of it residing primarily in the nonprocessing state, as supported by cryo-EM particle distributions^53^. Robust stimulation occurred only when NUB1 and FAT10 were added together (Fig. 4g and Supplementary Fig. 10b). Notably, the NTD-UBL fragment of NUB1 failed to stimulate ATPase activity, indicating that UBL domain binding itself has no allosteric effects. Instead, the observed stimulation likely results from active degradation and conformational shifts to substrate-processing states, with increased ATP hydrolysis and an open gate of the 20S core particle. Adding the NTD-UBL fragment together with the complementary N-terminal deletion variant NUB1^Δ1^^–^^175^ in complex with FAT10 also did not stimulate the proteasomal ATPase activity (Fig. 4g), ruling out an UBL-mediated proteasome activation for UBL-independent FAT10 turnover. The NUB1 UBL domain, thus, localizes the NUB1–FAT10 complex to the proteasome for FAT10 engagement and degradation, with no obvious allosteric effects, which also agrees with our structural data presented below.

### The NUB1 UBL domain binds to the proteasomal Rpn1

**Fig. 5:**
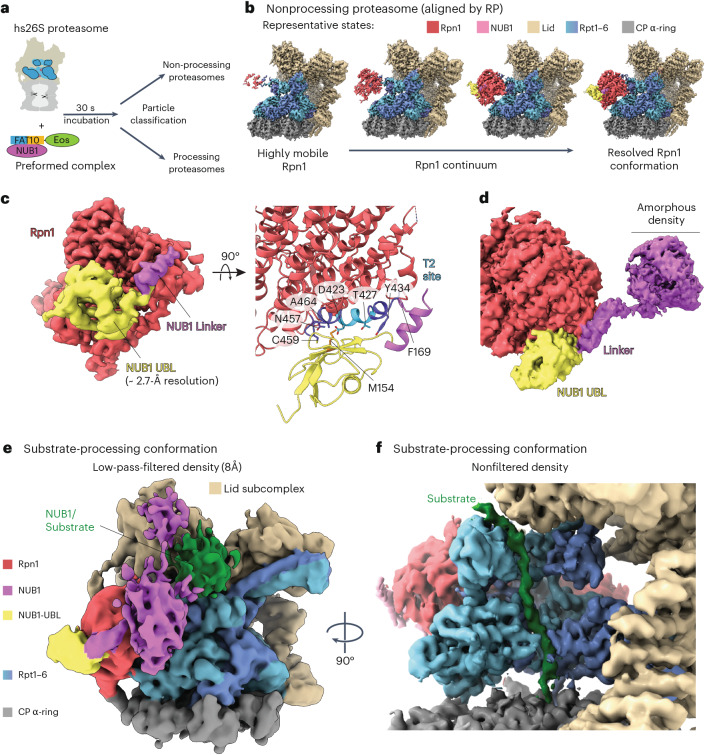
Structures of the NUB1–FAT10-bound *hs*26S proteasome. **a**, For the cryo-EM sample, the *hs*26S proteasome was mixed with the preformed NUB1–FAT10–Eos complex and incubated for 30 s before freezing. NUB1–FAT10–Eos-bound proteasome particles were then classified into nonprocessing and processing states. **b**, Representative states for the nonprocessing proteasome reveal the high flexibility of Rpn1 and consequently the Rpn1-bound NUB1–FAT10–Eos complex. Proteasomes were classified on the basis of Rpn1 conformations, showing a continuum of dynamic and more rigid states. This classification yielded a high-resolution reconstruction for the entire 19S regulatory particle (RP), Rpn1 (red) and the Rpn1-bound UBL domain of NUB1 (yellow). **c**, Left, local refinement of Rpn1 in the nonprocessing *hs*26S proteasome shows the NUB1 UBL domain (yellow) and the neighboring linker (purple) bound to Rpn1 (red). Right, local refinement of the Rpn1–NUB1^UBL^ portion of the nonprocessing *hs*26S proteasome particles allows unambiguous atomic modeling, showing the interactions of a β-sheet in the NUB1 UBL domain (yellow) with the T2 site of Rpn1 (red) and the docking of F169 in the NUB1 UBL linker (purple) with a hydrophobic pocket of Rpn1. **d**, Low-threshold representation revealing an amorphous, poorly resolved density (purple) that likely represents the flexible core body of NUB1 with bound FAT10–Eos. **e**, Low-pass-filtered (8 Å) cryo-EM map of the substrate-processing *hs*26S proteasome that was established by incubating proteasomes with NUB1 and FAT10–Eos for 60 s before freezing. Although highly dynamic and poorly resolved, NUB1 (purple) is still bound at this stage of substrate processing. A more distinct yet low-resolution density (green) at the entrance of the ATPase N-ring potentially represents the tough-to-unfold Eos moiety of the FAT10–Eos substrate after the FAT10 portion has been unfolded and translocated into the central channel. **f**, Nonfiltered density of the substrate-processing *hs*26S proteasome, rotated relative to the depiction in **e** by ~90° to the left and with the ATPase subunits Rpt4 and Rpt5 removed for a better view of the central channel. Substrate density continues through the AAA+ motor channel and into the 20S core particle (CP), indicating that likely the entire FAT10 portion of the FAT10–Eos substrate was unfolded and translocated at this stage of substrate degradation.

**Extended Data Fig. 6:**
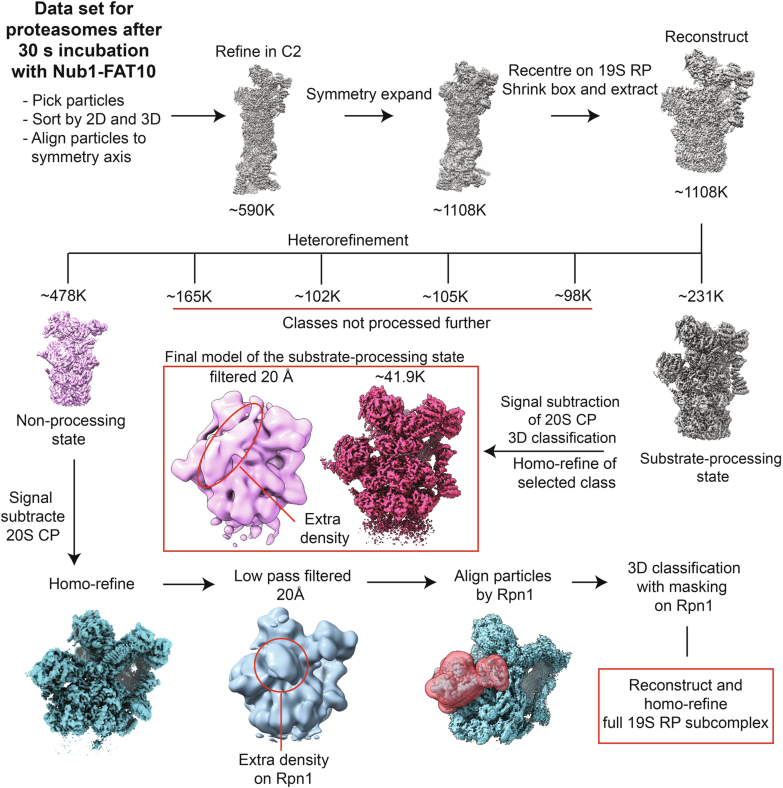
Processing workflow for the cryo-EM data set of proteasomes incubated with NUB1/FAT10 for 30 s. Preincubated FAT10-Eos (5 µM) and NUB1 (6 µM) were added to *hs*26S proteasome (2 µM) for 30 s before plunge freezing for Cryo-EM. The further classification of models for the non-processing proteasome is described in Figures E7 and S10. CryoSparc was used for all data processing and ChimeraX for visualization.

To determine the structural details of NUB1-mediated FAT10 delivery, we solved the cryo-EM structures of the ATP-hydrolyzing *hs2*6S proteasome either 30 s or 60 s after incubation with the preformed NUB1–FAT10–Eos complex. Consistent with active degradation, we observed the proteasome in processing states, where FAT10–Eos was engaged by the ATPase motor and partially threaded through the central channel, and in the nonprocessing, engagement-competent state with the NUB1–FAT10–Eos complex bound to the proteasome surface (Fig. 5a, Extended Data Fig. 6 and Table 1). Nonprocessing and processing conformations were easily distinguished on the basis of the major conformational transitions that occur upon substrate engagement and lead to a coaxial alignment of the N-ring, AAA+ ATPase ring and the 20S core particle^53–55^.

**Table 1:** Cryo-EM data collection, refinement and validation statistics

	Nonprocessing 26S with bound NUB1 and FAT10–Eos (EMD-42506, PDB 8USB)	Locally refined Rpn1-bound NUB1^UBL^ (EMD-42508, PDB 8USD)	NUB1–FAT10–Eos processing 26S proteasome (EMD-42507, PDB 8USC)
**Data collection and processing**			
Magnification	×81,000	×81,000	×81,000
Voltage (kV)	300	300	300
Electron exposure (e^–^/Å^2^)	50	50	50
Defocus range (μm)	−0.7 to −2.0	−0.7 to −2.0	−0.7 to −2.0
Pixel size (Å)	1.048	1.048	1.048
Symmetry imposed	*C*_1_	*C*_1_	*C*_1_
Initial particle images (no.)	1,108,000	1,108,000	231,000
Final particle images (no.)	50,000	372,000	19,200
Map resolution (Å)	2.7	2.7	3.1
FSC threshold	0.143	0.143	0.143
Map resolution range (Å)	2.7–4	2.7–4	3.1–5
**Refinement**			
Initial model used (PDB code)	7W37	AlphaFold model (rigid-body fit)	6MSK
Model resolution (Å)	3.2 (masked)	2.9 (masked)	3.6 (masked)
FSC threshold	0.5	0.5	0.5
Map sharpening *B* factor (Å^2^) (determined/applied)	46.4/not sharpened	85.9/not sharpened	39.7/not sharpened
Model composition	32	5	35
Nonhydrogen atoms	70,132	6,879	69,878
Protein residues	8,840	886	8,858
Ligands	Zn^2+^: 1		Zn^2+^: 1
	Mg^2+^: 6		Mg^2+^: 5
	ATP: 5		ATP: 3
	ADP: 1		ADP: 3
			UNK: 12
*B* factors (Å^2^) (min/max/mean)			
Protein	50.15/272.85/121.53	75.83/224.67/124.51	30.58/241.83/99.71
Ligand	66.26/133.67/86.61	–	46.60/164.37/77.25
Root-mean-square deviations			
Bond lengths (Å)	0.004	0.003	0.003
Bond angles (°)	0.547	0.671	0.526
Validation			
MolProbity score	1.96	1.91	2.75
Clashscore	9.95	11.11	58.60
Poor rotamers (%)	2.57	1.86	0.61
Ramachandran plot			
Favored (%)	97.31	97.24	91.38
Allowed (%)	2.66	2.64	8.17
Disallowed (%)	0.03	0.11	0.44

UNK, unknown amino acid.

**Extended Data Fig. 7:**
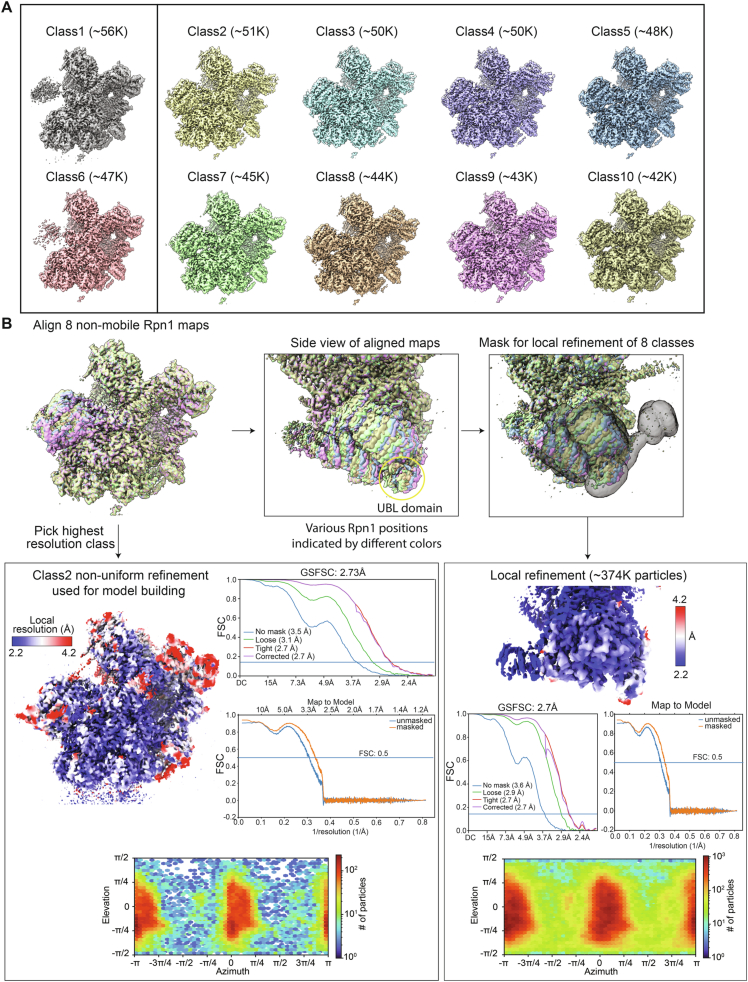
Continued cryo-EM data processing for the 30 s data set, focusing on Rpn1 classification. **a)** Using Rpn1-masked classification and refinement, 10 models of the non-processing *hs*26S proteasome are shown with particle numbers. Models 1 and 6 (left) show a blurry Rpn1 with continuous density, while the other 8 exhibit a resolved Rpn1 yet in slightly varied positions. **b)** An alignment of all Rpn1-resolved classes highlights these subtle differences in Rpn1 and therefore NUB1’s UBL position. The highest resolution class of the 19S RP subcomplex was further refined and used to build an atomic model (bottom left), for which the Gold-Standard FSC plot, map-to-model FSC plot, and distribution of particle orientations are show. Since NUB1’s UBL domain was at slightly lower resolution, further local refinement was performed on the Rpn1-NUB1 UBL interaction with all 8 Rpn1-resolved classes (bottom right), also showing the Gold-Standard FSC plot, map-to-model FSC plot, and distribution of particle orientations.

**Extended Data Fig. 8:**
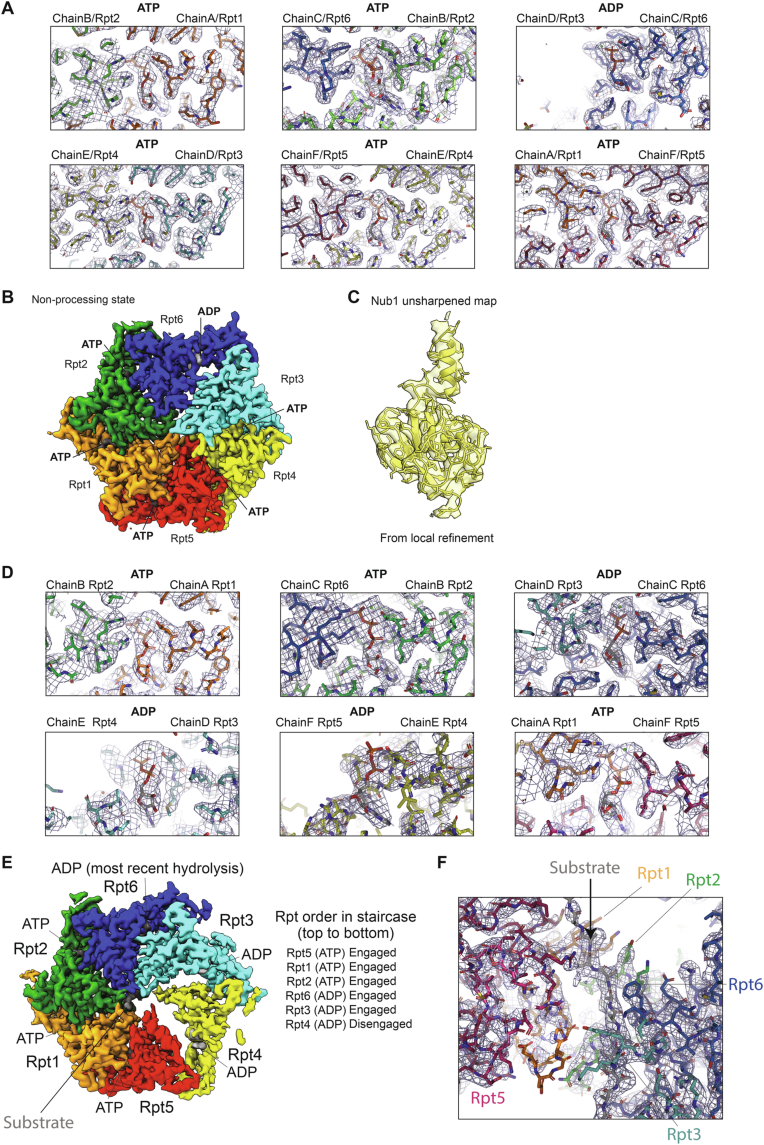
Examples of the well-resolved density for the ATPase motor and NUB1’s UBL domain from the 30 s data set of the non-processing *hs*26S proteasome. **a)** Atomic model focused on the nucleotide binding sites of Rpt1–Rpt6 in unsharpened maps generated by non-uniform refinement of Class2 non-processing proteasomes. The name of each chain/ATPase subunit and its clockwise-next neighbor in the ATPase ring are shown above the maps on the right and left side, respectively. Rpt1 is depicted in orange, Rpt2 in green, Rpt6 in blue, Rpt3 in cyan, Rpt4 in yellow, and Rpt5 in red. **b)** Top view of ATPase motor density colored as in A) with nucleotide positions and identity indicated. **c)** NUB1’s UBL domain density derived from a locally refined model. **d)** Atomic model focused on the nucleotide binding sites of Rpt1–Rpt6 in unsharpened maps. The name of each chain/ATPase subunit and its clockwise-next neighbor in the hexameric ATPase ring are shown above the maps on the right and left side, respectively. Rpt1 is depicted in orange, Rpt2 in green, Rpt6 in blue, Rpt3 in cyan, Rpt4 in yellow, and Rpt5 in red. **e)** Top view of ATPase motor density colored as in D) with nucleotide positions and identity indicated. The Rpts are arranged in a spiral staircase, with Rpt5 at the top, Rpt3 at the bottom, and Rpt4 as the mobile seam subunits on its way to the top. Rpt1, 2, 6, and 3 are engaged with the substrate polypeptide, whereas Rpt4 is disengaged and Rpt5 has not yet engaged the substrate. The ADP in Rpt6 likely originated from the most recent ATP-hydrolysis event, which precedes the disengagement of Rpt3 from the substrate and the substrate re-engagement of Rpt4 at the top of the staircase. **f)** Side view of the translocating substrate polypeptide contacted within the central channel by the pore-1 loop tyrosine of multiple Rpt subunits.

In the nonprocessing state, the NUB1–FAT10–Eos complex showed only weak, unresolvable density that likely originates from an intrinsic flexibility within the NUB1 complex itself and from its binding to Rpn1, which is the most mobile subunit of the 19S regulatory particle (Fig. 5b, Extended Data Figs. 6 and 7 and Supplementary Fig. 11). 3D classification focused on Rpn1 yielded ten structures with resolutions for the ATPase motor ranging from 2.5 to 3 Å and Rpn1 adopting variable positions (Extended Data Fig. 7). We chose one representative high-resolution model (~2.5 Å; Fig. 5b and Extended Data Fig. 7), which reveals the ATPase hexamer with five ATPs and one ADP, in Rpt6 (Extended Data Fig. 8a–c). Local refinement of Rpn1 produced a 2.7–3-Å map that enabled atomic modeling of the NUB1 UBL domain bound to the Rpn1 T2 site (Fig. 5c and Extended Data Fig. 7b). Similar to the deubiquitinase Usp14, whose UBL domain binds the T2 site in a slightly varied position^56^, the NUB1 UBL domain uses a hydrophobic center (M154 and L156) that is flanked by charged residues to interact with Rpn1 (Fig. 5c and Supplementary Fig. 12).

Interestingly, there is an additional anchor point between NUB1 F169, located in the linker following the UBL domain, and a pocket on Rpn1 (Fig. 5c and Supplementary Fig. 11), possibly orienting NUB1 during FAT10 delivery (Fig. 5c and Supplementary Fig. 12). However, despite extensive 3D classifications, variability analyses and refinements of the 19S regulatory particle, we were unable to resolve the NUB1 core, UBA1–UBA3 and the bound FAT10 (Fig. 5d and Supplementary Fig. 11). We assume that these domains get splayed out when NUB1 binds to the proteasome or during FAT10 release for engagement, and high mobility of the NUB1 core may be essential for the FAT10 UBL1 domain to sample different positions during its insertion into the ATPase channel. Ubiquitinated substrates are bound to the proteasome with similarly high flexibility before their engagement by the ATPase motor, which has so far prevented their visualization by cryo-EM.

**Extended Data Fig. 9:**
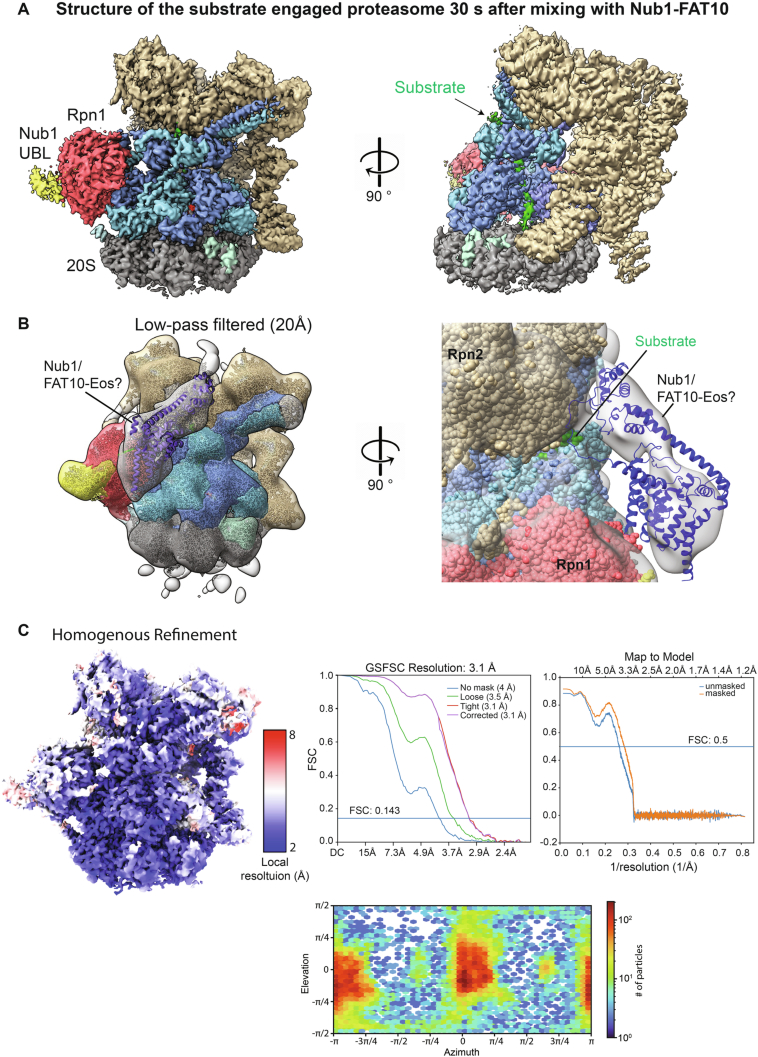
High-resolution model of the substrate-processing *hs*26S proteasome from the 30 s data set. **a)** Left: Reconstruction used for generating the atomic model, with Rpn1 shown in red, NUB1’s UBL domain in yellow, the ATPase motor in alternating blue and cyan, the 20S CP in gray, the lid subcomplex in sand color, and the substrate in green. Right: Reconstruction rotated by 90 degrees and with the density for the Rpt4 and Rpt5 ATPase subunits removed to visualize the translocating substrate density inside the central channel. **b)** A low pass filtered model of the density in A), shown for the entire top part of the proteasome (left) and focused on the space between Rpn1 and Rpn2 after a 90 degree rotation (right) to visualize the large extra density that connects to the well-resolved UBL domain of NUB1 and the translocating substrate. The size of this density agrees well with the size of NUB1, as indicated by the roughly docked structural model for NUB1’s core and UBA domains (purple ribbon presentation). However, NUB1 could not be well resolved, as it is likely present in multiple different positions and with variable orientations of its domains relative to each other. **c)** Local resolution, GSFSC curve, map-to-model FSC, and distribution of particle orientations for the substrate-processing model of the *hs*26S proteasome.

**Extended Data Fig. 10:**
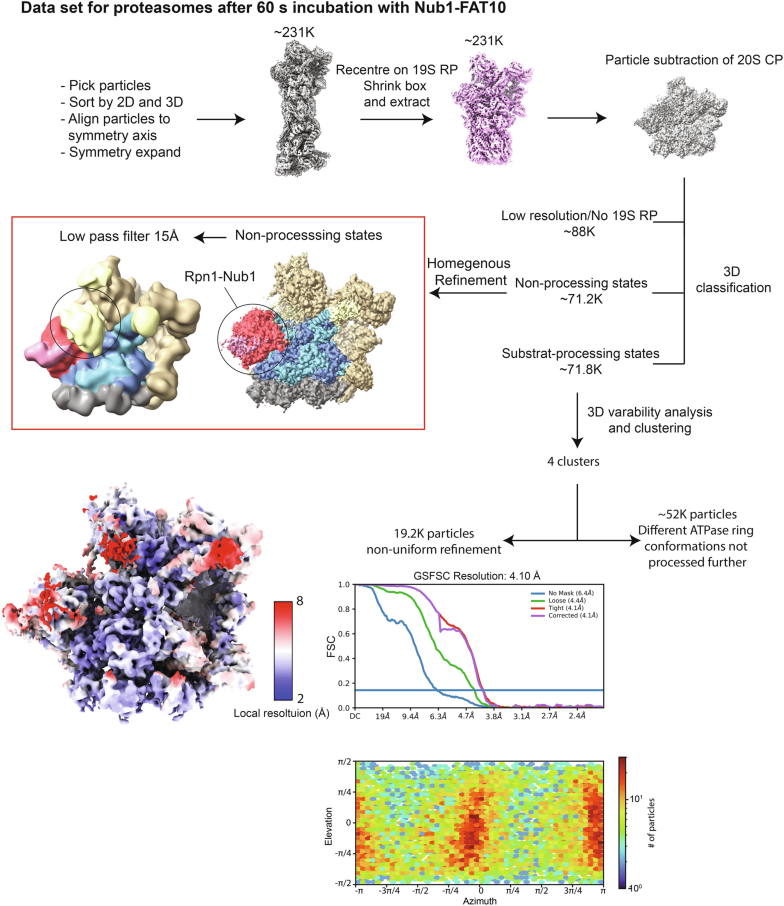
Cryo-EM data processing workflow for the 60 s data set of substrate-processing *hs*26S proteasome. A preincubated mixture of FAT10-Eos (5 µM) and NUB1 (6 µM) was added to *hs*26S proteasome (2 µM) for 60 s before plunge freezing for cryo-EM. The derived model shows an overall similar conformation as the substrate-processing state after 30 s incubation, albeit at lower resolution and with more density at the entrance to the central processing channel. Shown at the bottom are the local resolution, GSFSC curve, and distribution of particle orientations. CryoSparc was used for all data processing, with ChimeraX for visualization.

The processing conformations of the NUB1–FAT10–Eos-bound *hs*26S proteasome at both the 30-s and 60-s time points showed substrate density extending through the N-ring, through the ATPase ring and into the 20S degradation chamber (Fig. 5e,f and Extended Data Figs. 8–10). The length of this substrate trace suggests that, in addition to the unfolded UBL1 domain of FAT10, UBL2 was pulled into the central channel. At the entrance to the N-ring, we observed unresolvable yet more defined globular density that may represent the tough-to-unfold Eos domain of the FAT10–Eos fusion. Interestingly, the NUB1 UBL domain remained bound to Rpn1 at this stage of substrate processing (Fig. 5e and Extended Data Fig. 9), indicating NUB1 retention at the proteasome after FAT10 unfolding and threading. Outside of the NUB1 UBL domain stably interacting with Rpn1, there appear no other contacts persisting long enough for high-resolution observation by cryo-EM (Extended Data Fig. 9). This is unlike the deubiquitinase Usp14, which uses additional interactions between its catalytic ubiquitin-specific protease (USP) domain and the ATPase ring to enable substrate deubiquitination^56–58^.

## Discussion

**Fig. 6:**
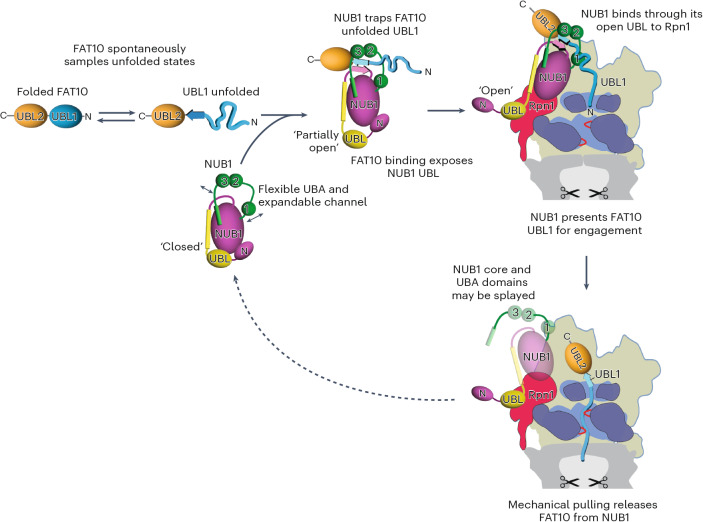
Model for the NUB1-mediated delivery of FAT10-ylated substrates to the 26S proteasome. Left, the low thermodynamic stability of FAT10 allows it to sample partially unfolded states. Middle, NUB1 uses a flexible linker that lines an internal channel within NUB1 to form a short antiparallel β-sheet with the last β-strand in the FAT10 UBL1 domain and thereby trap an unfolded state. This complex formation is rate limiting for FAT10 degradation by the proteasome, as it depends on spontaneous FAT10 unfolding. Right, trapping of FAT10 causes the UBL domain of NUB1 to adopt a partially open conformation, which enables the interaction of this UBL domain with the T2 site of the proteasomal Rpn1 subunit and the presentation of the unfolded UBL1 domain of FAT10 for engagement by the ATPase motor. The core and UBA domains of NUB1 may subsequently splay out for FAT10 release, yet NUB1 appears to stay bound to Rpn1 during FAT10 degradation before dissociating for another round of FAT10 substrate delivery.

Here, we determined a previously unknown mechanism for substrate delivery to the *hs*26S proteasome, in which NUB1 functions as an ATP-independent chaperone to trap FAT10 in a partially unfolded state, recruits it to the 19S regulatory particle through Rpn1 binding and presents the FAT10 unstructured N terminus for engagement by the ATPase motor (Fig. 6). This mechanism parallels the Ufd1–Npl4-mediated unfolding of polyubiquitinated substrates by the AAA+ unfoldase p97 (Cdc48 in yeast), whereby ubiquitin serves in targeting and as an initiation region after its unfolding through Npl4 binding^15–17^. Similarly, the FAT10 UBL1 domain provides both the recruitment and the initiation signals, enabling the degradation of any FAT10-ylated protein by the 26S proteasome in an NUB1-dependent manner. By contrast, ubiquitinated substrates require intrinsic unstructured initiation regions for proteasomal engagement. Because the FAT10 UBL1 domain enters the proteasome first, the position of the FAT10 conjugation site determines the starting point for unfolding and translocation on a substrate, requiring the proteasome to potentially process a branch point and thread two strands through the central channel. The inability to reconstitute FAT10 conjugation in vitro has limited our understanding of this process, underscoring the need for future studies to investigate FAT10-ylated substrate degradation in detail.

Isolated FAT10 or FAT10-ylated proteins are not inherently susceptible to robust proteasomal degradation, relying on the interferon-inducible NUB1 cofactor for regulatory control. This dependence may allow fine-tuned turnover of numerous substrates involved in cell-cycle control, nuclear factor-κB (NF-κB) activation, DNA damage response, autophagy and mitophagy^59–63^. Alternatively, there may be other mechanisms to circumvent this dependence on NUB1, for instance, through the ubiquitination of FAT10 and its delivery to p97. Interestingly, NUB1-mediated FAT10 degradation also has similarities to the recently identified delivery of transcription factor substrates by the midnolin cofactor for proteasomal turnover. There, the catch domain of midnolin captures a β-strand of the substrate for delivery to the proteasome through an unknown mechanism^18^.

Our biochemical, biophysical and structural studies revealed an interesting mode of complex formation between NUB1 and FAT10, whereby a looped-out portion of NUB1 forms an antiparallel β-sheet with the last β-strand of the FAT10 UBL1 domain, trapping it in an unfolded state. Essential and apparently rate determining for the complex formation is the spontaneous unfolding of the UBL1 domain. It remains unclear whether the unfolded UBL1 domain of FAT10 inserts itself into the looped-out portion of NUB1 or whether the NUB1 C-terminal helices detach from the core domain to transiently open the loop for capture of FAT10. The unfolded UBL1 of FAT10 is then presented to the ATPase motor and, upon engagement and mechanical pulling, FAT10 is either released by NUB1 splaying out its domains or threaded through the NUB1 looped-out region. Our cryo-EM studies suggested that NUB1 remains bound to Rpn1 after the FAT10 portion of the FAT10–Eos fusion substrate is translocated, although this may also reflect binding of new, potentially uncomplexed NUB1 because of the high concentrations in the sample. Importantly, NUB1 is highly dynamic and, therefore, not resolvable, possibly because its domains detach from each other and adopt various different states when bound to the proteasome. NUB1 uses its UBL domain to interact with the Rpn1 T2 site, similar to the deubiquitinase Usp14, yet it lacks additional persisting contacts to further stabilize the NUB1–FAT10 complex. The high mobility is likely important for allowing the unstructured N-terminal UBL1 domain of FAT10 to enter the central channel of the ATPase motor.

In summary, we identified NUB1 trapping of the unfolded UBL1 domain of FAT10 as a key mechanism for substrate delivery and proteasomal engagement. Future studies should address whether NUB1 can similarly trap NEDD8 or other β-strand-containing proteins for processing by the 26S proteasome or p97. The high promiscuity of this NUB1-mediated substrate turnover and FAT10 expression in immune cells upon inflammation, viral infection and in multiple cancers could make the specific FAT10-ylation of neosubstrates for proteasomal degradation an attractive alternative to the proteolysis-targeting chimera (PROTAC) technology, which typically relies on small-molecule-induced ubiquitination and frequently requires p97 to prepare well-folded proteins for proteasomal engagement.

## Methods

### Cloning

All truncations were made using NEBuilder HiFi DNA assembly master mix (M5520AVIAL, New England Biolabs) or using Q5 mutagenesis (E0555L, New England Biolabs). Point substitutions were introduced using Q5 PCR mutagenesis.

The NUB1 sequence was synthesized for codon-optimized *E.* *coli* expression as three double-stranded DNA (dsDNA) fragments (Integrated DNA Technologies) and assembled into a pGEX-6P-1 vector using NEBuilder HiFi DNA assembly master mix. The final expressed protein is GST–3C–NUB1, whereby the glutathione *S*-transferases (GST) can be removed by precision protease leaving a GPGS overhang at the N terminus. NUB1L was also cloned in the same way as NUB1.

#### Amino acid sequence for wild-type NUB1

MAQKKYLQAKLTQFLREDRIQLWKPPYTDENKKVGLALKDLAKQYSDRLECCENEVEKVIEEIRCKAIERGTGNDNYRTTGIATIEVFLPPRLKKDRKNLLETRLHITGRELRSKIAETFGLQENYIKIVINKKQLQLGKTLEEQGVAHNVKAMVLELKQSEEDARKNFQLEEEEQNEAKLKEKQIQRTKRGLEILAKRAAETVVDPEMTPYLDIANQTGRSIRIPPSERKALMLAMGYHEKGRAFLKRKEYGIALPCLLDADKYFCECCRELLDTVDNYAVLQLDIVWCYFRLEQLECLDDAEKKLNLAQKCFKNCYGENHQRLVHIKGNCGKEKVLFLRLYLLQGIRNYHSGNDVEAYEYLNKARQLFKELYIDPSKVDNLLQLGFTAQEARLGLRACDGNVDHAATHITNRREELAQIRKEEKEKKRRRLENIRFLKGMGYSTHAAQQILLSNPQMWWLNDSNPETDNRQESPSQENIDRLVYMGFDALVAEAALRVFRGNVQLAAQTLAHNGGSLPPELPLSPEDSLSPPATSPSDSAGTSSASTDEDMETEAVNEILEDIPEHEEDYLDSTLEDEEIIIAEYLSYVENRKSATKKN*

NUB1 constructs cloned in this study (truncations and mutants) are based on NUB1 numbering for the mentioned sequence. Construct residue numbers are as follows: UBA1–UBA3 domains, 376–528; NUB1 NTD domain, 1–72; UBL domain, 75–161; NTD-UBL, 1–158; linker-trap domain, 159–601; linker-trap domain, 175–601; trap domain, 229–601; ΔUBA1–3, Δ379–527, ΔUBA1–3 + linker, Δ379–527 with insertion at deleted position 4× TGS; ΔUBA1, Δ376–412; ΔUBA2–3, Δ422–527; ΔUBA2–3 + linker, Δ422–527 with insertion at deleted position 4× TGS; Δbs-linker, 211–223. Substitutions introduced into NUB1 constructs included Y572A, D278A, N279A, L562S/I565S, R414A/L418A, D214P/D216P and C317A/R324A. Insoluble NUB1 constructs constituted residues 1–372, 228–372, 239–532, 376–601 and 376–601. Substitutions that gave insoluble constructs included the NTD-UBL fragment with M154R and L156R and full-length NUB1 with L256D and L260D.

FAT10 was synthesized codon-optimized for *E.* *coli* expression and cloned into pCDB179 (Addgene, plasmid 91960) by Gibson assembly. For labeling with sortase, GG was added to the N terminus of the wild-type FAT10 sequence by Q5 mutagenesis, whereby a GG scar is left after Smt3 cleavage with His–ULP1. His–Smt3 cysteine-less GG–FAT10 (FAT10^C0^, with C7T, C9T, C134L, C160S and C162S) was gene-synthesized and subcloned into a pET28 vector by GeneArt (Thermo Fisher Scientific). GG–FAT10 was used for comparison to GG–FAT10^C0^, GG–FAT10^H75A^, GG–FAT10^H75D^ and GG–FAT10^H75K^ in assays. For all other experiments involving unlabeled FAT10, the wild-type FAT10 sequence displayed below was used.

#### Amino acid sequence for wild-type FAT10

MAPNASCLCVHVRSEEWDLMTFDANPYDSVKKIKEHVRSKTKVPVQDQVLLLGSKILKPRRSLSSYGIDKEKTIHLTLKVVKPSDEELPLFLVESGDEAKRHLLQVRRSSSVAQVKAMIETKTGIIPETQIVTCNGKRLEDGKMMADYGIRKGNLLFLACYCIGG*

To create Eos3.2 constructs, Ub_4_–Eos3.2–intein–CBD (chitin-binding domain) and Ub_4_–Eos3.2–tail–intein–CBD from a previous publication^45^ were used as templates. For FAT10–Eos, FAT10–Eos–tail, FAT10^ΔUBL2^–Eos, FAT10^ΔUBL2^–Eos–tail, FAT10^ΔUBL1^–Eos and FAT10^ΔUBL1^–Eos–tail, the wild-type FAT10 vector (His–Smt3–FAT10) was linearized and PCR fragments of Eos–intein–CBD or Eos–tail–intein–CBD were inserted by HiFi assembly. To generate UBL-fused Eos constructs, FAT10–Eos and FAT10–Eos–tail vectors were used as templates for PCR amplification, whereby FAT10 was removed and subsequently replaced by HiFi assembly with either the NUB1 UBL domain (amplified from the full-length codon-optimized NUB1 sequence) or NEDD8 (ordered as dsDNA from Integrated DNA Technologies, codon-optimized for *E.* *coli*).

The plasmid for expression of His–ULP1 was from Addgene (plasmid 64697), the His–sortase plasmid was created as previously described^4^ and the His–GST–3C plasmid and purified His–TEV (tobacco etch virus) protease were sourced from QB3 MacroLab (University of California, Berkeley (UCB)).

### Protein expression in *E.coli*

All proteins were expressed in *E.* *coli* BL21* grown at 37 °C with 200 rpm in Terrific broth medium (24 g of yeast extract, 20 g of tryptone and 8 ml of glycerol, buffered with phosphate pH 7.2). After reaching an optical density at 600 nm of 0.6–0.8, cells were cooled to ~16 °C, expression was induced with IPTG (0.25 mM) and cells were left growing overnight at 16 °C.

### NUB1 purification

After overnight expression at 16 °C, all NUB1-expressing cells were harvested and suspended in lysis buffer (60 mM HEPES pH 7.4, 325 mM NaCl, 25 mM KCl, 5% (v/v) glycerol, 10 mM MgCl_2_ and 0.5 mM TCEP) supplemented with EDTA-free protease inhibitor tablets (11836170001, Roche) and benzonase (70664, Novagen). Cells were lysed by sonication on ice and clarified at 15,000*g* for 45 min at 4 °C. Lysates were then flowed slowly (~1 ml min^−1^) multiple times over glutathione (GSH) resin (16101, Thermo Fisher Scientific) that was pre-equilibrated with lysis buffer, before successive washes (at least ten column volumes) with lysis buffer and suspension in two column volumes of lysis buffer. GST–3C protease was added for overnight incubation at 4 °C before collection of the flowthrough and another two column volumes of washes, clarification (4,000*g*, 15 min), concentration using an Amicon Ultra-15 30-kDa-cutoff concentrator (fUFC905008, MilliporeSigma) and gel filtration using a Superdex (SD) 200 increase 10/300 column or SD200 16/600 column, depending on scale and yields of protein. Fractions containing NUB1 were collected from a single peak, concentrated to ~10–15 mg ml^−1^ and snap-frozen as single-use aliquots (10 µl) in liquid N_2_ for storage at −80 °C. Protein concentration was estimated using the absorbance at 280 nm (*A*_280_) and all NUB1 proteins had *A*_280/260_ ratios of 0.5–0.6.

### FAT10 purification

His–Smt3–FAT10-expressing cells were harvested and suspended in lysis buffer supplemented with benzonase, EDTA-free protease inhibitor tablets, 300 mM NaCl and 20 mM imidazole. Cells were sonicated and clarified before flowing lysate over pre-equilibrated Ni-NTA resin several times. Contaminants were removed by several successive washes with lysis buffer, before incubation of resin with His–ULP1 protease overnight in lysis buffer supplemented with 150 mM NaCl. The cleavage reaction was not mechanically moved and instead resuspended with a pipette a few times before leaving the reaction overnight at 4 °C. The reaction was typically mixed one more time before moving to room temperature for 10 min. After this, flowthrough containing cleaved FAT10 was collected for anion exchange. FAT10 was carefully diluted with lysis buffer (usually about tenfold in volume) before binding to a HiTrap SP HP column (17115201, Cytiva) and elution with a linear gradient of 0–1,000 mM NaCl. FAT10 eluted as a single peak, which was concentrated using an Amicon Ultra-15 10-kDa-cutoff concentrator and clarified by centrifugation (20,000*g* at 4 °C) before fractionation with an SD75 increase 10/300 column or SD75 16/600 column. FAT10 eluted as a single peak and was concentrated to ~10 mg ml^−1^, before flash-freezing as single-use 10-µl aliquots and storage at −80 °C. All FAT10 mutants were purified like the wild type, except for the single UBL1 domain of FAT10 that skipped the cation exchange and His–Smt3–FAT10 fusions that were eluted with lysis buffer supplemented with 250 mM imidazole.

### Purification of UBL–Eos3.2

All UBL–Eos3.2 constructs were expressed as fusion proteins with an N-terminal His–Smt3 and C-terminal intein–CBD. Cells were lysed in lysis buffer supplemented with benzonase, EDTA-free protease inhibitor tablets and 20 mM imidazole, before sonication, clarification and binding to Ni-NTA resin. After extensive washing with lysis buffer, proteins were eluted with lysis buffer supplemented with 250 mM imidazole and bound to chitin resin (S6651L, New England Biolabs), before washing in lysis buffer and overnight incubation with lysis buffer supplemented with 200 mM DTT and His–ULP1 protease. The flowthrough was collected and flowed by gravity over Ni-NTA resin to remove His–ULP1 and His–Smt3 proteins before concentration and gel filtration using an SD200 16/600 column in GF buffer. Protein concentrations were estimated from *A*_507_ and *A*_280_. Because not all Eos3.2 matures, we used the concentration estimated from *A*_280nm_ as the concentration of UBL–Eos3.2 substrates.

### Purification of sortase, His–ULP1 and His–GST–3C

His–sortase A and His–ULP1 were purified with identical conditions. After suspension in lysis buffer supplemented with benzonase, 200 mM NaCl and 20 mM imidazole, cells were lysed by sonication and clarified. Lysate was flowed over Ni-NTA resin by gravity, the resin was washed extensively and proteins were eluted with lysis buffer supplemented with 250 mM imidazole and 150 mM NaCl. For His–GST–3C protease, conditions were similar except that lysate was flowed over GSH resin before washing and elution with 20 mM GSH in lysis buffer. Eluted His–GST–3C was subsequently bound to a HiTrap Q HP column (17115401, Cytiva) and eluted over a linear gradient (0–1,000 mM NaCl). All proteins were concentrated and fractionated using an SD200 16/600 column in 30 mM HEPES pH 7.4, 150 mM NaCl, 5% (v/v) glycerol and 0.5 mM TCEP. Proteins were concentrated to ~10 mg ml^−1^ (estimated by *A*_280_) and frozen in liquid N_2_ for storage at −80 °C.

### *hs*26S proteasome purification from HTBH–Rpn11 expressing HEK293 cells

The HEK293 cells expressing the hexahistidine, TEV cleavage site, biotin and hexahistidine (HTBH)-tagged *hs*26S proteasomes were previously generated^64^ and a gift from L. Huang. Cells were adapted for suspension to increase scale and ultimately yields of *hs*26S proteasome. For adaptation, cells were grown by gradually lowering FBS (16000044, Thermo Fisher Scientific) concentration from 10% to 5% (v/v) on plates and, after three passages, were grown in FreeStyle 293 expression medium (12338018, Thermo Fisher Scientific) with 2% (v/v) FBS. Cells were harvested and moved to a shaker flask, where the suspension cells were grown at 8% CO_2_, 37 °C with 120 rpm shaking in FreeStyle 293 expression medium with 2% (v/v) FBS. Cells were passaged twice a week at 5 × 10^5^ and newly thawed cells were grown with puromycin.

For expression and purification, 4 l of HTBH–Rpn11 HEK293 cells were grown for 72 h after passaging to 5 × 10^5^ and harvested by centrifugation at 4,000*g*. Cell pellets were resuspended in lysis buffer supplemented with benzonase, EDTA-free proteasome inhibitor tablets, 0.01% NP-40 and 2 mM ATP. Cells were lysed with a Dounce homogenizer (usually 15×) followed by sonication on ice with low amp (20%) and 10 s on and off for 2 min. Lysates were clarified by centrifugation at 15,000*g* for 30 min at 4 °C and flowed over pre-equilibrated Pierce high-capacity streptavidin agarose (3 ml of resin). After two rounds of binding, beads were carefully washed five times with 3 ml of lysis buffer (+2 mM ATP) before suspension in 3 ml of lysis buffer (+2 mM ATP). TEV protease (250 µg) was added and resin was incubated at room temperature for 60 min or overnight at 4 °C. Flowthrough from resin was collected, beads were washed with two additional column volumes and collected for concentration to ~250 µl with an Amicon Ultra-15 100-kDa-cutoff concentrator. The sample was clarified by centrifugation at 20,000*g* for 15 min at 4 °C before fractionation with an S6 increase 10/300 column in GF buffer supplemented with 2 mM ATP. Fractions containing 26S proteasomes were pooled and concentrated to ~50–100 µl before freezing as 10-µl single-use aliquots in liquid N_2_ and stored at −80 °C. The concentration of samples was measured by Bradford reagent with BSA as a standard and we assumed that the *hs*26S proteasome was 2,600 kDa for molar calculations.

### Purification of *sc*26S proteasome and *sc*20S proteasome

The *S.* *cerevisiae* 20S core and 26S holoenzyme were purified from the yAM54 or YYS40 yeast strains, respectively. Yeast were grown in yeast extract–peptone–dextrose medium at 30 °C for 3 days before harvesting. Yeast were lysed by freezing in lysis buffer with liquid N_2_ and using a 6875 freezer mill dual chamber cryogenic grinder (SPEX Sample Prep). The 3×Flag–Pre1 yeast cells were resuspended in 60 mM HEPES pH 7.6, 500 mM NaCl, 0.1% (v/v) NP-40 and 5% glycerol (v/v); the 3×Flag–RPN11 cells were lysed in 60 mM HEPES pH 7.4, 25 mM NaCl, 25 mM KCl, 5% (v/v) glycerol, 10 mM MgCl_2_ 0.5 mM TCEP and 2 mM ATP. Proteasomes were bound to M2 anti-Flag resin (Sigma). For 20S particle preparations, the resin was washed with 1 M NaCl to remove bound RP. Otherwise, the 26S proteasome was washed in low-salt buffer with 60 mM HEPES pH 7.4, 25 mM NaCl, 25 mM KCl, 5% (v/v) glycerol, 10 mM MgCl_2_, 0.5 mM TCEP and 2 mM ATP. The 20S core particle or proteasomes were eluted from Flag resin using 0.3 mg ml^−1^ 3×Flag peptide and further purified by SEC with a Superose6 Increase 10/300 column (GE) equilibrated with 60 mM HEPES pH 7.4, 25 mM NaCl, 25 mM KCl, 10 mM MgCl_2_ 2, 5% (v/v) glycerol and 0.5 mM TCEP (for the 26S proteasome, 2 mM ATP was also added). Proteins are quantified using Bradford reagent with BSA as a protein standard.

### Degradation assays by SDS–PAGE

All SDS–PAGE degradation assays were performed at 30 °C in 0.65-ml microcentrifuge tubes (1605-0001, SealRite). Proteins were diluted with reaction buffer (60 mM HEPES pH 7.4, 25 mM NaCl, 25 mM KCl, 5% (v/v) glycerol, 10 mM MgCl_2_, 0.5 mM TCEP and 0.5 mg ml^−1^ BSA) supplemented with 5 mM ATP and 1× ATP regeneration mix (5 mM ATP, 0.03 mg ml^−1^ creatine kinase and 16 mM creatine phosphate). FAT10 alone or FAT10 with excess NUB1 were incubated in reaction buffer (at 2–4 times the final assay concentration) for at least 30 min on ice before diluting with buffer and mixing with 2× concentrated 26S proteasome stock. Concentrations used in specific assays are indicated in figure legends; however, for example, in Fig. 1b, NUB1 at 30 µM was incubated with FAT10 at 10 µM for 30 min on ice before mixing with 2× 26S proteasome (2× at 200 nM) to reach a final reaction volume of 10 µl with 100 nM 26S proteasome, 15 µM NUB1 and 5 µM FAT10. After incubation for an indicated amount of time, reactions were quenched with 10 µl of SDS×PAGE loading buffer (Tris pH 7, 20% (v/v) glycerol, 4% (w/v) SDS, 0.04 mg of brilliant blue and 200 mM DTT) and 12-µl aliquots were loaded onto SDS–PAGE gels, which were then Coomassie stained.

### Multiturnover FAT10–Eos3.2 degradation assays

For multiturnover reactions, FAT10–Eos3.2 was in excess over the *hs*26S proteasome and specific concentrations of each component are given in the figure legends. Degradation of Eos3.2 was monitored by SDS–PAGE or fluorescence loss using a BMG Labtech CLARIOstar plate reader with MARS software at 30 °C through emission at 520 nm after excitation at 500 nm. Proteins were diluted in prewarmed reaction buffer with 5 mM ATP and preformed FAT10–Eos3.2 + NUB1 complexes were mixed 1:1 with 26S proteasomes to reach final concentrations. For Michaelis–Menten kinetics, the initial rate of signal loss at each FAT10–Eos3.2 concentration was determined by linear regression and converted to degradation rate (substrate per enzyme per min), before plotting and calculating *k*_cat_ and *K*_m_ using GraphPad 10.2.3. (Prism). Individual replicates were processed and the means ± s.d. of the *k*_cat_ and *K*_m_ are reported.

### Single-turnover kinetics

For single-turnover experiments, the unfolding rate of Eos3.2 was measured by incubation with excess *hs*26S proteasome and NUB1. For Fig. 1g, fluorescence loss of Eos3.2 was measured using a BMG Labtech CLARIOstar plate reader with MARS software at 30 °C and monitoring emission at 520 nm after excitation at 500 nm. Samples were first loaded at 2× concentrations in separate wells of a prewarmed 384-well plate. After 1-min incubation in the plate reader, 5 µl of *hs*26S proteasome (4 µM) was pipetted from one well to the substrate-containing well and mixed quickly before starting the read, such that the first 6–8 s were usually not recorded. Reactions were typically monitored for ~20 min before fitting single or double exponentials using GraphPad (Prism). The determined rate constants for the single-exponential (*k*) or first phase of the double-exponential decay (*k*_fast_) were used to calculate the normalized unfolding rate (substrate per enzyme per min) and the means ± s.d. of three experiments are reported for each condition.

### Sortase labeling

His–sortase at 25 µM was incubated with FAT10 (with GG at its N terminus) at 30 µM for 30 min at room temperature with 5-FAM conjugated to the N terminus of a peptide (HHHHHHLPETGGG, ordered from Biomatik) at 500 µM in 1× reaction buffer without BSA, supplemented with 10 mM CaCl_2_ and 1 mM DTT. Labeled FAT10 (^FAM^FAT10) proteins were enriched using Ni-NTA resin followed by spin filtering through 0.22-µm spin columns and fractionation using a SD75 increase 10/300 column. ^FAM^FAT10 concentrations were estimated using FAM absorbance.

### Binding assays with ^FAM^FAT10

All binding assays were performed in 10-μl volumes in a preheated (30 °C) 384-well black plate (Costar) using a BMG Labtech CLARIOstar plate reader with MARS software. Polarization of ^FAM^FAT10 was measured through the emission at 535 nm after excitation at 480 nm. To measure the *K*_d_ value between FAT10 and NUB1, ^FAM^FAT10 was incubated at 10× final concentration, either alone or with increasing NUB1 concentrations for 30 min on ice, before diluting to 1× (100 nM ^FAM^FAT10 plus NUB1 at indicated concentrations) and measuring polarization. To measure kinetics of binding, ^FAM^FAT10 at 2× concentration (40 nM) was mixed with NUB1 at 2× concentration and polarization was measured over time. Single exponentials were fit to each curve, observed rates constants were plotted against the NUB1 concentration and *k*_on_ was determined by linear regression. For binding experiments at single concentrations, 2× ^FAM^FAT10 (200 nM) was preincubated with 2× NUB1 (10 µM) for 30 min on ice before measuring polarization and taking the end point after a stable polarization reading. GraphPad (prism) was used for data analysis.

### ATPase assays

ATP hydrolysis rates were determined in an assay coupled to reduced NADH, where *A*_340_ was monitored over time in a BMG Labtech CLARIOstar plate reader with MARS software at 30 °C. The 1× ATPase mix (5 mM ATP, 3 U per ml of pyruvate kinase (Sigma), 3 U per ml of lactate dehydrogenase (Sigma), 1 mM NADH and 7.5 mM phosphonyl pyruvate (Sigma)) was incubated with the *hs*26S proteasome (typically at 100 nM) in the presence or absence of FAT10 (10 µM) and NUB1 (15 µM) in reaction buffer. When using FAT10 + NUB1, proteins were preincubated on ice for at 20 min. ATPase rates were measured using the linear part of the curve.

### Peptidase assays

Stocks of each reagent were prepared at 4× final concentrations, LLVY–AMC (400 µM), *hs*26S proteasome stock (400 nM), FAT10 (60 µM) and NUB1 (60 µM), in reaction buffer supplemented with 5 mM ATP. For *hs*26S proteasome stocks, 4× ATP regeneration mix was also supplemented; in conditions with ATPγS, the analog was added at 20 mM to the 4× *hs*26S stock. FAT10 and NUB1 were diluted 1:1 with buffer or each other to form a complex on ice for 30 min, making a 2× substrate stock. Samples (prewarmed to 37 °C) were mixed in the following order: FAT10 (or NUB1 or FAT10 + NUB1) followed by LLVY–AMC and *hs*26S proteasome to initiate the reaction. AMC fluorescence was measured at 445 nm after excitation at 345 nm in a preheated 384-well black plate (Costar) using a BMG Labtech CLARIOstar plate reader with MARS software. The linear part of the reaction was analyzed by linear regression and converted to a percentage relative to the 26S proteasome alone.

### SEC and SEC–MALS

NUB1 (50 µM) was incubated with FAT10 (75 µM) for 30 min on ice before fractionating with an SD200 Increase 10/300 column. A single peak for the NUB1–FAT10 complex was pooled and SEC–MALS was conducted on an Agilent Technologies 1100 series with a 1260 Infinity lamp, Dawn Heleos II and the Optilab T-Rex (Wyatt Technologies), using an SD200 Increase 10/300 column. The column was equilibrated with 60 mM HEPES pH 7.4, 50 mM NaCl, 50 mM KCl, 5% glycerol, 10 mM MgCl_2_ and 0.5 mM TCEP with a flow rate of 0.5 ml min^−1^. The same was repeated for NUB1 alone.

### SEC–SAXS

Data were collected at SIBYLS beamline by coupling an SAXS flow cell with an online Agilent 1260 Infinity high-performance LC system using a Shodex KW803 column^65^. The column was equilibrated at a flow rate of 0.65 ml min^−1^ with 60 mM HEPES pH 7.4, 150 mM NaCl, 5% glycerol and 0.5 mM TCEP. NUB1 (50 μM), FAT10 (55 μM), NUB1 + FAT10 (50 μM + 55 μM) and NUB1^ΔUBL^ + FAT10 (50 μM + 55 μM) samples were loaded one by one onto SEC and SAXS data were collected with 2-s X-ray exposures for a 25-min elution. Data were collected on a Pilatus3 2M pixel array detector with the following parameters: wavelength, 1.232 Å; camera length, 2 m; *q* measurement range, 0.01–0.45; temperature, 22 °C. Data were processed using RAW^66^ and FoXS^66,67^.

### HDX–MS sample preparation

Samples for FAT10 and NUB1 were diluted to 10× stock concentration and four stocks of protein were prepared: FAT10 (10 µM), FAT10 + NUB1 (10 µM + 15 µM), NUB1 (10 µM) and NUB1 + FAT10 (10 µM + 15 µM). Samples were left on ice for at least 30 min. Replicates for each set of HDX–MS experiments were performed with three different preparations of FAT10 and NUB1. Labeling buffer was prepared as a 10× stock by diluting 300 mM HEPES pH_read_ 7.0 (effectively pD 7.4), 250 mM NaCl, 250 mM KCl, 100 mM MgCl_2_ and 5 mM TCEP in 100% D_2_O; an equivalent H_2_O (10× stock) buffer was made at pH 7.4. A 2× quench buffer was prepared with 200 mM glycine pH 2.4, 3.5 M guanidinium chloride and 200 mM TCEP. Each sample was prepared by diluting 2 µl in 18 µl of D_2_O labeling buffer and incubated for an indicated amount of time at 25 °C before quenching (rapidly mixing 20 µl, 1:1 with quench buffer), flash-freezing in liquid N_2_ and storage at −80 °C. An unlabeled sample was prepared as above except diluted into H_2_O buffer before quenching.

### HDX–MS

Samples (in a random order) were immediately thawed and injected one by one into a cooled valve system (Trajan LEAP) coupled to an LC system (Thermo UltiMate 3000) maintained at 2 °C. Proteins were digested on the column by flowing quenched samples at 200 μl min^−1^ in 0.1 % formic acid over in-house prepared protease columns (inner diameter, 2 mm; length, 2 cm; IDEX C-130B) at 10 °C. The proteases, aspergillopepsin (Sigma-Aldrich, P2143) and porcine pepsin (Sigma-Aldrich, P6887), were crosslinked to POROS Al aldehyde-activated resin (Thermo Fisher Scientific, 1602906) in that order. Peptides were desalted for 4 min with POROS R2 reversed-phase resin (1112906, Thermo Fisher Scientific) hand-packed into a trap column (inner diameter, 1 mm; length, 2 cm; IDEX C-128) at 2 °C. Subsequently, peptides were separated using a C8 column (72205-050565, Thermo Fisher Scientific, BioBasic-8; particle size, 5 μm; inner diameter, 0.5 mm; length, 50 mm) at a flow rate of 40 μl over 14 min with a 5–40% gradient of 100% acetonitrile and 0.1% formic acid followed by 90% over 30 s. Peptides were eluted directly into a Q Exactive Orbitrap MS instrument operating in positive mode (resolution, 70,000; automatic gain control (AGC) target, 3 × 10^6^; maximum injection time (IT), 50 ms; scan range, 300–1,500 *m*/*z*). Before all subsequent injections, protease columns were washed twice with 100 μl of 1.6 M guanidinium chloride and 0.1% formic acid. All LC and MS methods were performed using Xcalibur 4.1 control software (Thermo Fisher Scientific). The analytical and trap columns were subject to sawtooth washes and equilibrated at 5% of 100% acetonitrile and 0.1% formic acid. For undeuterated samples and each condition (FAT10, FAT10 + NUB1, NUB1 and NUB1 + FAT10), a separate MS/MS experiment was run to identify peptide lists using the MS settings described, except for the following: resolution, 17,500; AGC target, 2 × 10^5^; maximum IT, 100 ms; loop count, 10; isolation window, 2.0 *m*/*z*; normalized collision energy, 28; charge state, 1 and ≥7 excluded; dynamic exclusion, 15 s.

Byonic (Protein Metrics) was used to identify FAT10 and NUB1 peptides from MS/MS spectra. Peptide lists (sequence, charge state and retention time) were exported from Byonic and imported into HDExaminer 3 (Sierra Analytics). When multiple peptide lists were obtained, all were imported and combined in HDExaminer 3. HDX–MS peptides were analyzed using HDExaminer 3, where peptide isotope distributions and deuteration amounts were calculated and extracted. For NUB1, all peptides were analyzed using unimodal analysis, whereas a large majority of the mass spectra for FAT10 peptides were fit with a bimodal, which calculated two centroid peaks and, therefore, two deuterated levels. We did not consider intensities of each peak because of mixed EX1–EX2 kinetic regimes for deuterium uptake, making accurate fitting ambiguous when peaks overlapped. We looked for the presence or absence of bimodal distributions and described an overarching effect from NUB1 binding. Uptake plots were fit from experimentally calculated deuterated levels for each peptide at each time point and Wood’s plots displaying all peptides were generated by extracting data from HDExaminer and plotting with Jupiter notebook using Python and Matplotlib. For FAT10, we generated the Wood’s plot by only considering the left peak (lightest peak) with comparison to unimodal peaks or, when present, the left peaks for the bimodal obtained from data for FAT10 + excess NUB1.

### Cryo-EM sample preparation and data collection

Samples were diluted in 20 mM HEPES pH 7.4, 25 mM NaCl, 25 mM KCl, 5 mM MgCl_2_, 2 mM ATP, 2.5% glycerol and 0.02% NP-40 as 2× stocks and centrifuged at 15,000*g* for 15 min at 4 °C. Proteasomes (4 µM) were mixed 1:1 with preincubated (20 min) FAT10–Eos + NUB1 (10 µM + 12 µM) complexes (final concentrations of 1 µM, 5 µM and 6 µM) for 30 s and 60 s before cryo plunging. Samples (3.5 µl) were applied to glow-discharged (25 mA, 25 s) UltrAufoil R2/2 200-mesh Au grids (Q250AR2A, EM Sciences). Using a Vitrobot (Thermo Fisher Scientific), glow-discharged grids were placed at 100% humidity and samples were applied and immediately blotted for 2.5 s (blot force = 10) before plunge-freezing in liquid ethane.

Grids were clipped and transferred to a Titan Krios transmission EM instrument operated at 300 keV (Thermo Fisher Scientific) with an energy filter (GIF quantum) and equipped with Gatan K3 using SerialEM. Images were taken at a nominal magnification of ×81,000 (1.048 Å pixel size) in super-resolution mode with a defocus ranging from −0.5 to −1.7 µm using SerielEM^68^. We collected 50 frames per shot with a total electron dose of ~50 e^−^ per Å^2^ per s. A total of 20,565 videos were collected for the 30-s dataset and 19,128 videos for the 60-s dataset.

### Cryo-EM data processing

All micrographs were patch-motion-corrected with contrast transfer function estimation using cryoSparc (version 4.3.1)^69^. From the 30-s and 60-sdatasets, 20,565 and 19,128 corrected micrographs were subjected to blob picker. Picked particle blobs were extracted with a 720-pixel box binned by 2. Particles were subjected to multiple rounds of two-dimensional classification before taking a small subset of particles (~50,000) and generating four ab initio reconstructions, where a single 30S proteasome model with secondary-structure features was selected. The 30S proteasome ab initio model was used to seed multiple rounds of heterogeneous refinement (with 4–10 classes) with a binned pixel size of 128. Particles that reached Nyquist frequencies (when binned) in heterogeneous refinement were aligned by homogeneous refinement in *C*_2_ to a symmetry-aligned low-pass-filtered 30S proteasome model, which was aligned manually in UCSF Chimera^70^. Symmetry expansion was used to effectively double the number of particles, as we wanted to focus on features of the 19S regulatory particle. Particles were then shifted using volume alignment tools in cryoSparc to where the 19S regulatory particle was at the center of the box and particles were re-extracted with a box size of 280 pixels. Homogeneous reconstruction was used to generate a 19S regulatory particle model with just over half of the 20S core particle, followed by homogeneous refinement. The 19S regulatory particle model was used to seed multiple rounds of heterogeneous refinement, which resulted in some low-resolution classes, a few nonprocessing classes and a single class containing substrate-engaged proteasomes. For selected nonengaged and engaged proteasome particle stacks, the 20S core particle signal was removed by generating a mask and using particle subtraction. Subtracted particles were subject to homogeneous reconstruction and homogeneous refinement.

For substrate-engaged proteasomes, rounds of heterogeneous refinement were used again to separate out states, resulting in four major ATPase states, each of which was subjected to homogeneous refinement followed by nonuniform refinement^71^. The largest class of substrate-engaged proteasomes from the 30-s dataset was subjected to another round of 19S regulatory particle masked 3D classification and nonuniform refinement, resulting in one high-resolution representative state for the FAT10-engaged NUB1-bound *hs*26S proteasome, and subsequent model building. The above method was also used for the 60-s dataset, except the final classification step was omitted. Many unsuccessful attempts were made to resolve additional density found in models with NUB1–FAT10, including 3D variability analysis^72^, 3D classification, heterogeneous refinement and particle subtraction.

For particles in nonengaged proteasome stacks, Rpn1 was clearly flexible with extra density. We generated ten classes through Rpn1-masked 3D classification and performed homogeneous refinement on each class. This gave rise to ten nonengaged proteasome structures with Rpn1 in varied positions; the remaining 19S regulatory particle models appeared almost identical. Two structures with a total of 103,000 particles showed Rpn1 as completely mobile, where extra density could still be seen through low-pass-filtering models. However, we could not resolve additional structures, likely because of the continuous mobility of Rpn1. The other eight classes showed a well-resolved Rpn1 with a UBL domain attached at variable resolutions. The highest-resolution model was chosen for nonuniform refinement and this model was used for model building. In addition, the eight models were combined for local refinement for a single high-resolution model of the NUB1 domain bound to Rpn1, which also allowed further modeling of the NUB1 linker following the UBL domain. Using the combined stack, homogeneous refinement was again used to align particles but with a larger mask covering more extra density, followed by 3D classification (20 classes with filtering resolution to 15 Å) and homogeneous refinement of each class. Each class contained an amorphous mass attached to the UBL of NUB1; however, despite notable efforts, this density could not be resolved. We used one of these models to represent the model for how NUB1 dynamically moves relative to the 19S regulatory particle and its own UBL, likely sampling variable positions to help FAT10 engage the proteasome.

### Cryo-EM model building and visualization of structures

For nonengaged proteasome models, we used a previous structure (PDB 7W37)^56^ as a starting model with rigid-body fitting using Chimera. However, our high-resolution models allowed us to detect multiple register errors. We replaced several chains (A, B, C, D, F, U, V, W, X, Y, Z, a, b, c, d, f and g) using AlphaFold models^73^. We did not replace chains E, G, H, I, J, K, L, M and e. We deleted parts or most of chains N, O, P, Q, R, S and T. Coot was used to manually curate side-chain positions and secondary-structure differences from AlphaFold models^74^. The high-resolution data allowed us to build unmodeled sequences, such as the N terminus of Rpn1, which is sandwiched between the toroidal domain of Rpn1 and Rpt1. We were able to unambiguously assign nucleotide densities. For engaged proteasome models, we used another structure (PDB 6MSK)^56^ with rigid-body fitting and extensive remodeling with Coot. Real-space refinement in PHENIX was performed iteratively with model building in Coot^75,76^. Figures were generated using PyMOL (version 1.8, Schrödinger), UCSF Chimera and ChimeraX^77^. Local resolution was displayed for each structure using local resolution estimation in cryoSparc with a Fourier shell correlation (FSC) of 0.143 and Chimera with a colored surface. Low-pass-filtered models were generated in cryoSparc with volume tools.

### Statistics and reproducibility

In general, biochemical experiments included at least three technical replicates. Coomassie-stained gels are representative of several repeats.

### Reporting summary

Further information on research design is available in the Nature Portfolio Reporting Summary linked to this article.

## Online content

Any methods, additional references, Nature Portfolio reporting summaries, source data, extended data, supplementary information, acknowledgements, peer review information; details of author contributions and competing interests; and statements of data and code availability are available at 10.1038/s41594-025-01527-3.

## Supplementary information

Supplementary Information Supplementary Figs. 1–13 and Table 1.

Reporting Summary

Supplementary Data 1 Raw data for all biochemical measurements presented in Supplementary Figs. 1, 2, 3 and 10.

Supplementary Data 2 Pooled MS data for HDX–MS analysis of FAT10.

Supplementary Data 3 Pooled MS data for HDX–MS analysis of NUB1.

## Source data

Source Data Fig. 1 Raw data for all biochemical measurements.

Source Data Fig. 2 Raw data for all biochemical measurements.

Source Data Fig. 3 Raw data for all biochemical measurements.

Source Data Fig. 4 Raw data for all biochemical measurements.

Source Data Figs. 1, 2 and 4 and Extended Data Fig. 1 Images of all uncropped gels.

Source Data Extended Data Fig. 1 Raw data for biochemical measurements.

Source Data Extended Data Fig. 2 Raw data for biochemical measurements.

Source Data Extended Data Fig. 3 Raw data for biochemical measurements.

Source Data Extended Data Fig. 4 Raw data for biochemical measurements.

## Data Availability

All data generated or analyzed during this study are included in this manuscript and the Supplementary Information. Structural data are available from the Electron Microscopy Data Bank (EMDB) and the Research Collaboratory for Structural Bioinformatics Protein Data Bank (PDB) (EMD-42506 and PDB 8USB for the nonprocessing 26S proteasome, EMD-42507 and PDB 8USC for the substrate-processing 26S proteasome at 30 s after substrate addition and EMD-42508 and PDB 8USD for focused refinement of the proteasomal Rpn1 subunit with the bound UBL domain of NUB1). SEC–SAXS data for NUB1 can be found online (https://simplescattering.com/) under accession code XSPKOJGM. Any additional information required to reanalyze the data reported in this paper is available from the lead contact upon request. Source data are provided with this paper.
