# Supplementary material for: NUB1 traps unfolded FAT10 for ubiquitin-independent degradation by the 26S proteasome
Source: Nat Struct Mol Biol. 2025 Apr 11;32(9):1752–65. doi: 10.1038/s41594-025-01527-3 (PMC12285911; doi:10.1038/s41594-025-01527-3)
Supplement: Supplementary file 1 — Supplementary Figs. 1–13 and Table 1. [file 41594_2025_1527_MOESM1_ESM.pdf]

nature structural & molecular biology

Supplementary information

https://doi.org/10.1038/s41594-025-01527-3

NUB1 traps unfolded FAT10 for ubiquitin-independent degradation by the 26S proteasome

In the format provided by the authors and unedited

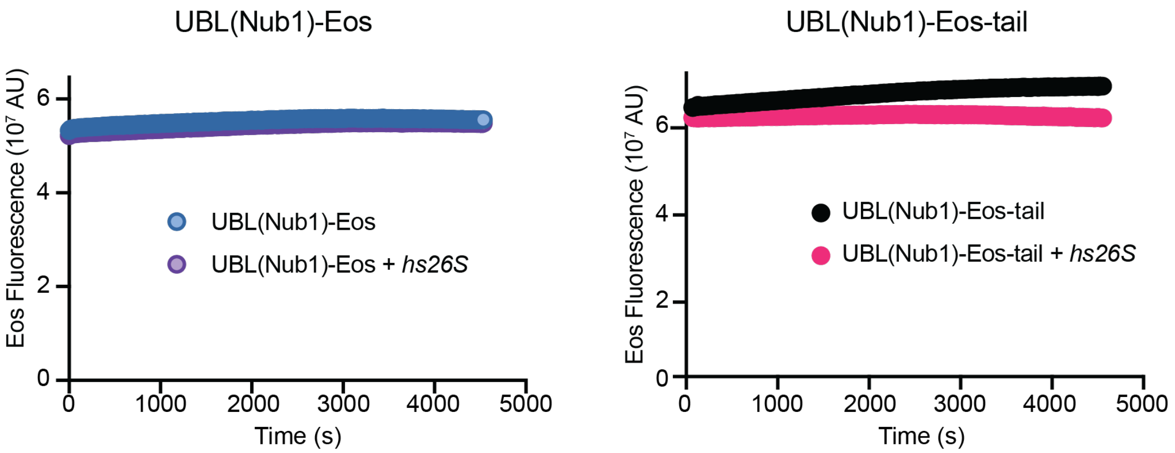

Supplementary Figure 1: NUB1's UBL domain is not sufficient for delivering a substrate for degradation. Example fluorescence traces for the incubation of the UBL(NUB1)-Eos fusion substrate (5 µM, left) or the UBL(NUB1)-Eos-tail fusion substrate (5 µM, right) with *hs*26S proteasome (100 nM).

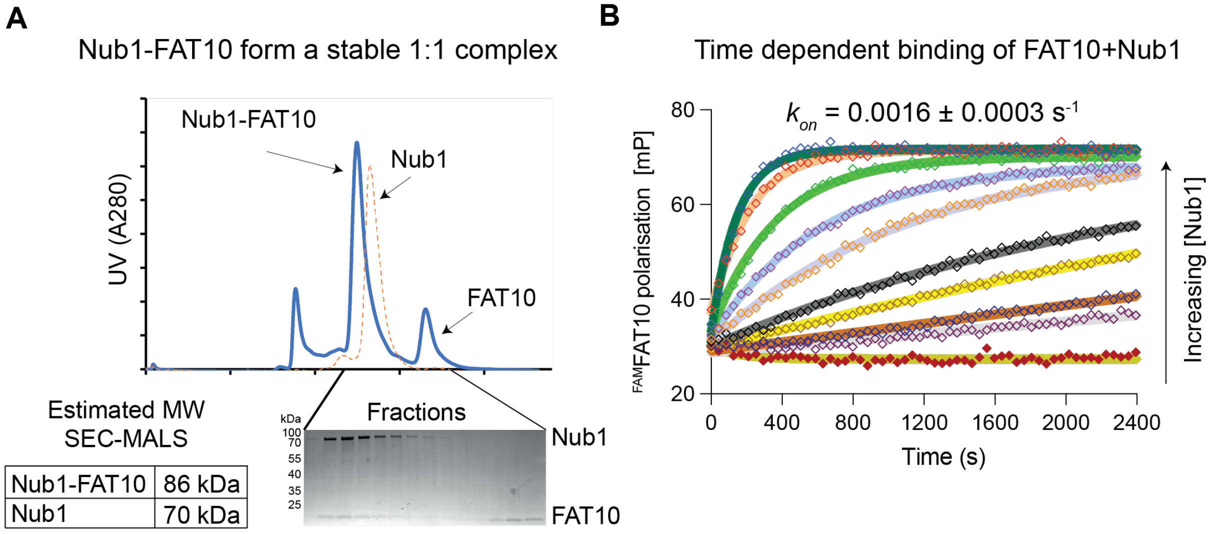

Supplementary Figure 2: NUB1 and FAT10 slowly form a high-affinity 1:1 complex. A) Elution profiles for the size-exclusion chromatography of NUB1 alone (dotted orange line) or a sample in which NUB1 (50 µM) was preincubated with FAT10 (75 µM) for 60 min. The Coomassie-stained SDS-PAGE gel below shows samples of the individual fractions. The void peak likely originates from unfolded FAT10 and subsequent aggregation. **B)** Time courses for the fluorescence polarization of ^{FAM}FAT10 (20 nM) after mixing with NUB1 at increasing concentrations (0, 16, 32, 63, 125, 250, 500, 1000, 2000 and 4000 nM).

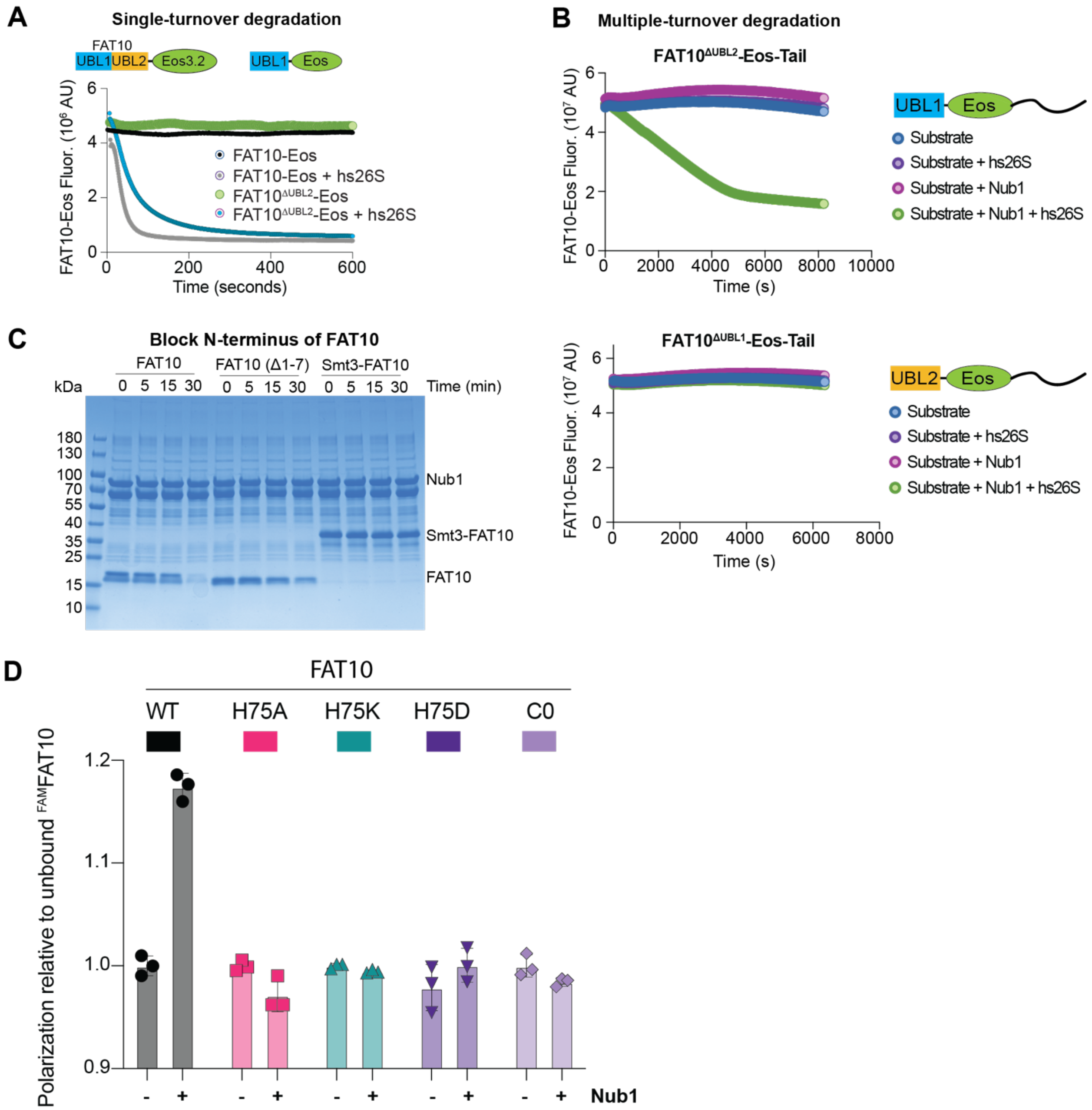

Supplementary Figure 3: UBL1 is necessary and sufficient for NUB1-mediated degradation of FAT10. A) Single-turnover degradations of FAT10-Eos and FAT10$^{\Delta UBL2}$-Eos (100 nM) by *hs*26S proteasome (2 µM) in the presence of NUB1 (10 µM) indicate that FAT10's UBL1 is sufficient for degradation, but the UBL2 domain is likely involved in forming a more productive complex with the proteasome for FAT10 turnover. **B)** Fluorescence-based assay for the degradation of FAT10$^{\Delta UBL2}$-Eos-tail (5 µM, top) and FAT10$^{\Delta UBL1}$-Eos-tail (5 µM, bottom) by *hs*26S proteasome (100 nM) in the absence and presence of NUB1 (15 µM). NUB1 and FAT10's UBL1 domain are required for FAT10 delivery and degradation, even in the presence of a long initiation region. **C)** Coomassie-stained gel to analyze the degradation of FAT10 or a His-Smt3-FAT10 fusion (10 µM) by *hs*26S proteasome (100 nM) in the presence of NUB1 (5 µM). The lack of His-Smt3-FAT10 degradation indicates that FAT10's free N-terminus is critical for insertion into and engagement by the proteasomal ATPase motor. Correspondingly, FAT10 degradation is

slowed down upon truncation of the flexible N terminus in FAT10 (Δ1-7). For complete gel image see Suppl. Fig. 13A. **D)** Fluorescence polarization measurements analyzing the complex formation between wild-type or mutant ^{FAM}FAT10 (100 nM) and NUB1 (10 μM), which were mixed and incubated for 30 mins on ice prior to the measurements. Shown are the mean values and standard deviations for N = 3 technical repeats, normalized to the values for free ^{FAM}FAT10.

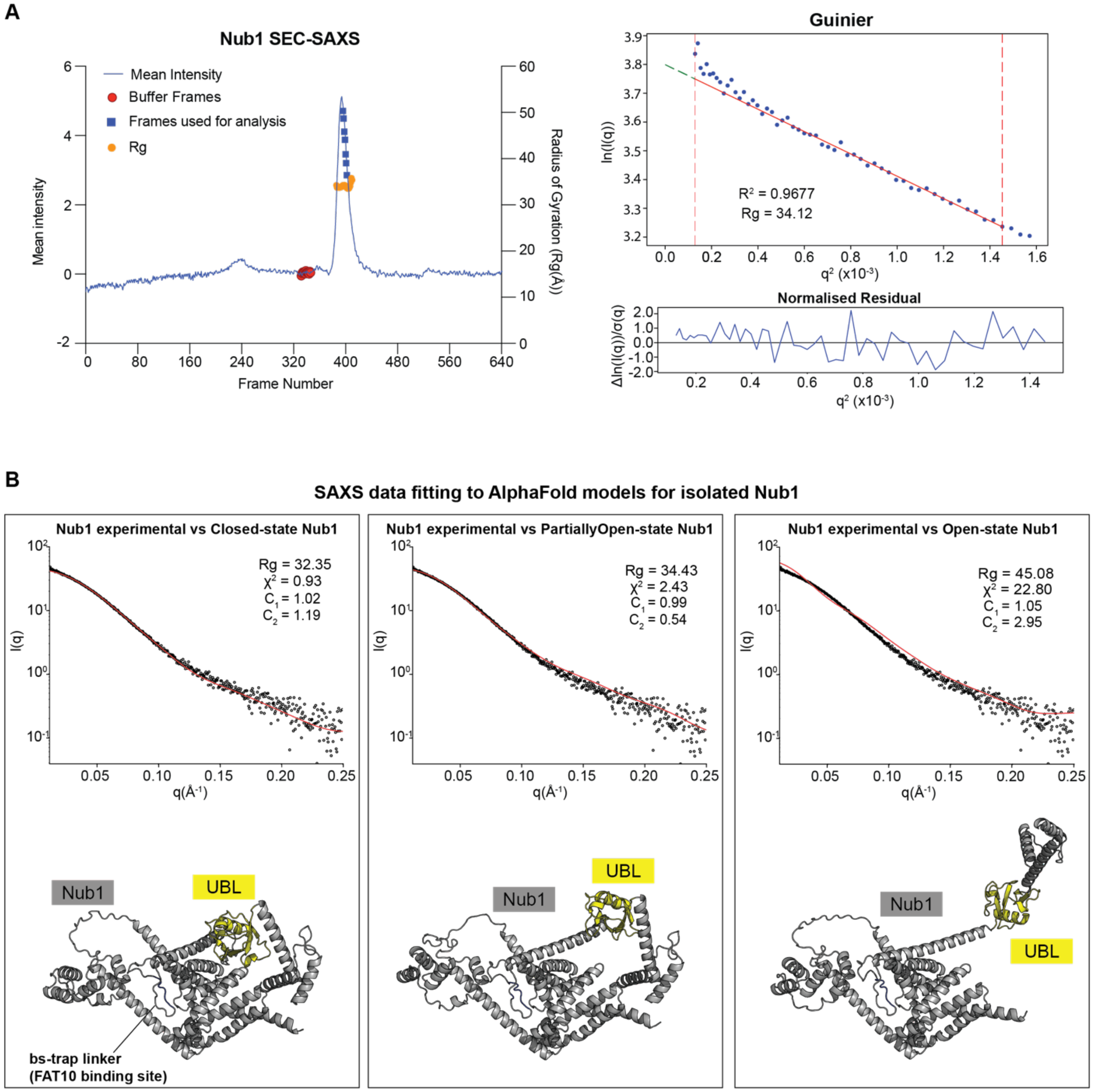

Supplementary Figure 4: Comparison of solution-state SAXS measurements with AlphaFold models of NUB1 indicates that the UBL domain is in a predominantly closed conformation. **A)** Left: NUB1 SEC-SAXS trace with the frames used for buffer subtraction and analyses indicated by red dots and blue squares, respectively. Right: Guinier plot for the buffer-subtracted averaged SAXS frames. **B)** Fit of experimental SAXS data to AlphaFold-generated models for closed, partially open, and open NUB1, using FOXS [66,67].

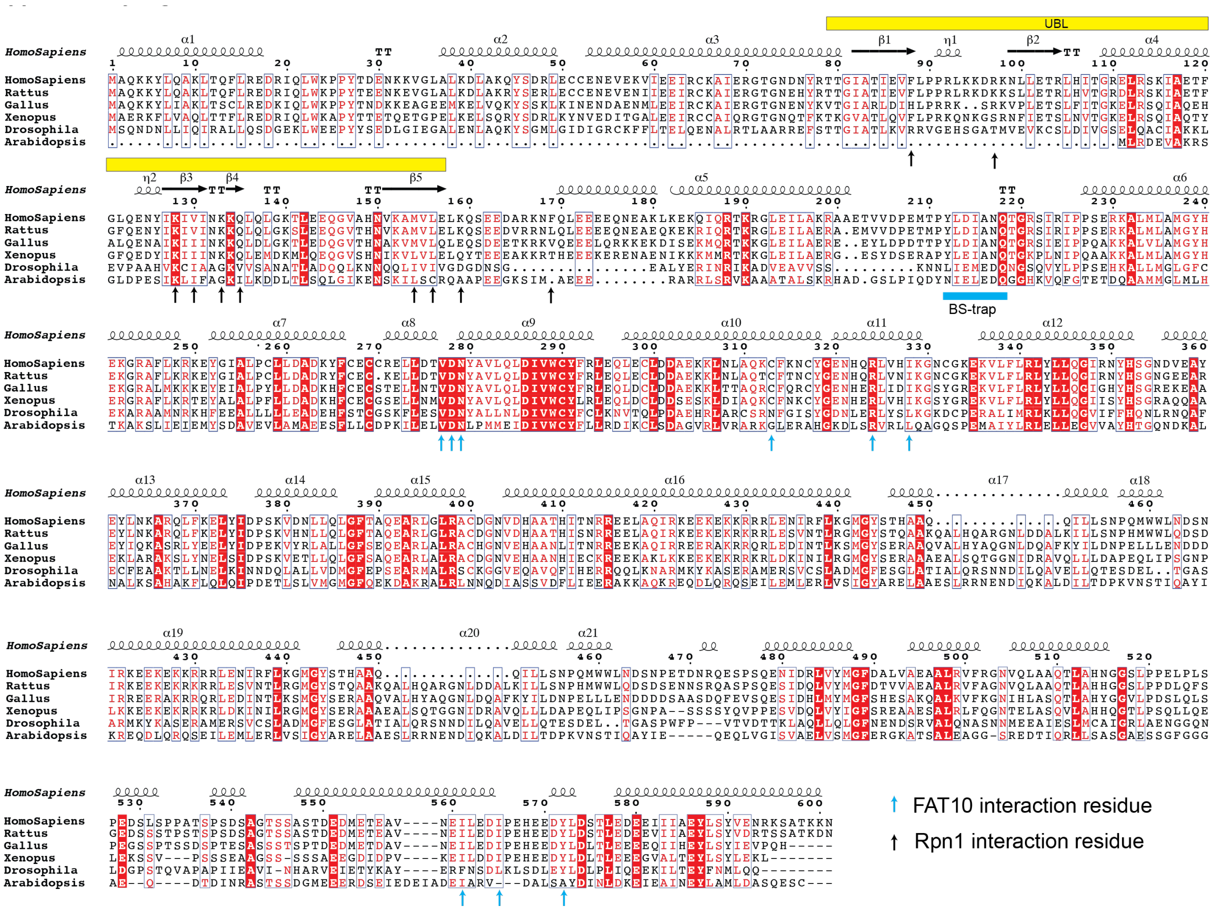

Supplementary Figure 5: Multiple sequence alignment of NUB1 amino acid sequences from model organisms. NUB1 is conserved in evolution from mammals to plants, unlike FAT10 which is only found in mammals. Clustal omega was used to align the Uniprot sequences, and the schematic was generated with ESPript [68] (https://espript.ibcp.fr) based on the AlphaFold model of NUB1 and input multiple sequence alignments.

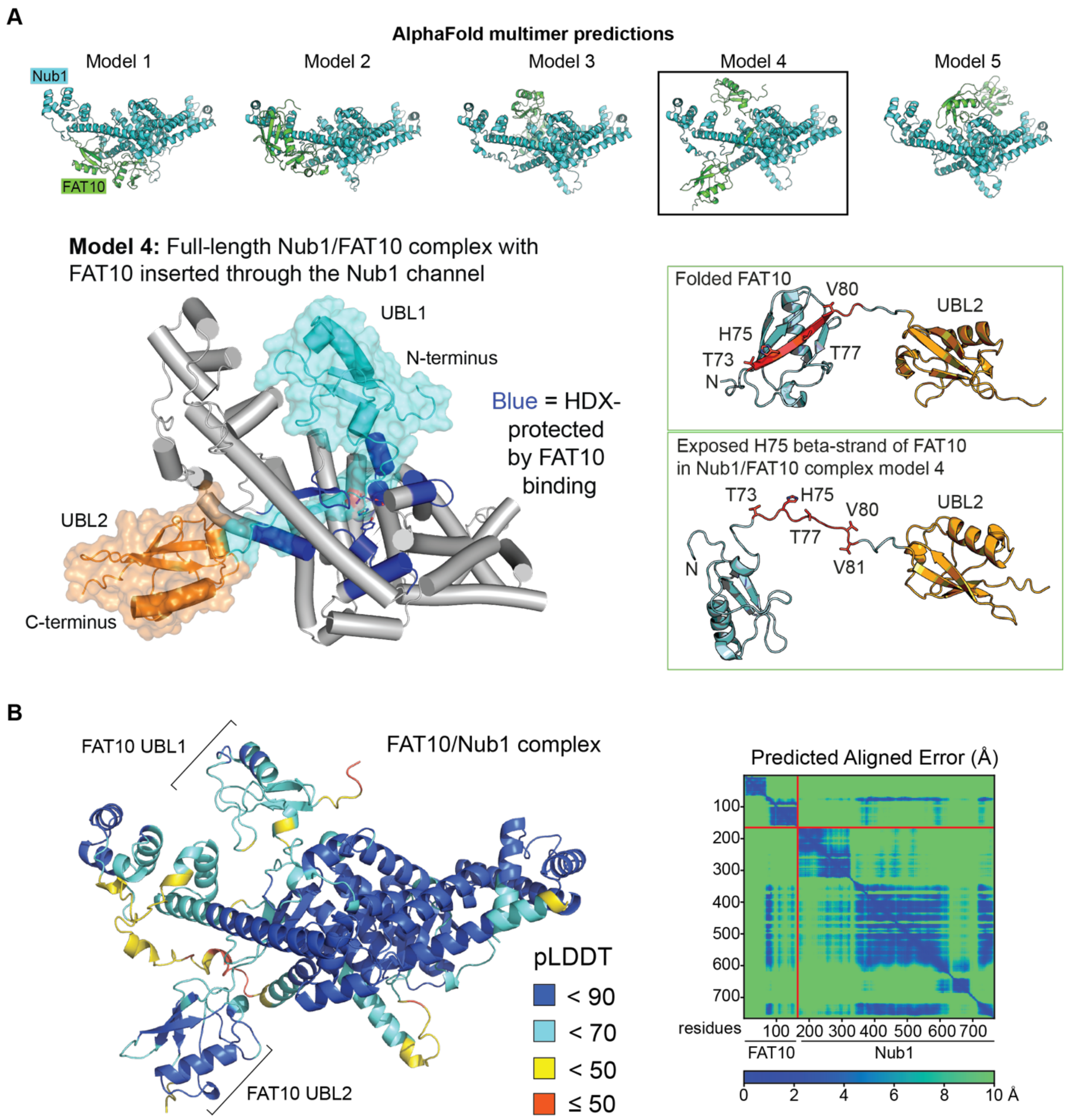

Supplementary Figure 6: AlphaFold-multimer model of the full-length NUB1/FAT10 complex. A) Top: AlphaFold-multimer generated a range of models with FAT10 (green) in various conformations bound to Nub1 (blue). Most were not confident based on the predicted aligned error (PAE) or not consistent with our results from biochemical and HDX-MS experiments. However, one model was consistent with those data and showed FAT10 inserted through the NUB1 channel, with the H75 beta-strand of FAT10 forming an anti-parallel beta sheet with beta-strand (BS)-trap linker of NUB1 (bottom left), similar to the structural model obtained with truncated NUB1 and FAT10 (Figure 3C). Bottom right: Comparison of folded FAT10 and FAT10 in the NUB1/FAT10 structural model 4 with unfolded UBL1 domain and exposed H75 beta strand. **B)** Left: pLDDT scores mapped onto the full-length NUB1/FAT10 complex structural

model 4. Right: PAE scores for the complex. Of note, the UBL domain of NUB1 remains docked to the core body of NUB1 in this model, however, this is inconsistent with our HDX-MS data that suggest release and exposure of this UBL domain upon FAT10 binding. This is likely an artifact of AlphaFold-multimer, which adds weights to domain-domain and protein-protein interactions, giving rise to predictions where interfaces might be correct, but not necessarily in the right context. NUB1's UBL domain likely binds to and releases from the core domain, and this equilibrium is shifted in the presence of FAT10. In addition, a partial beta-grasp fold is predicted for FAT10's UBL1 domain, which may exist at times, but HDX-MS suggest a completely labile protein. The UBL1 domain of NUB1-bound FAT10 most likely lacks stable secondary structure elements that would normally slowly exchange in HDX experiments.

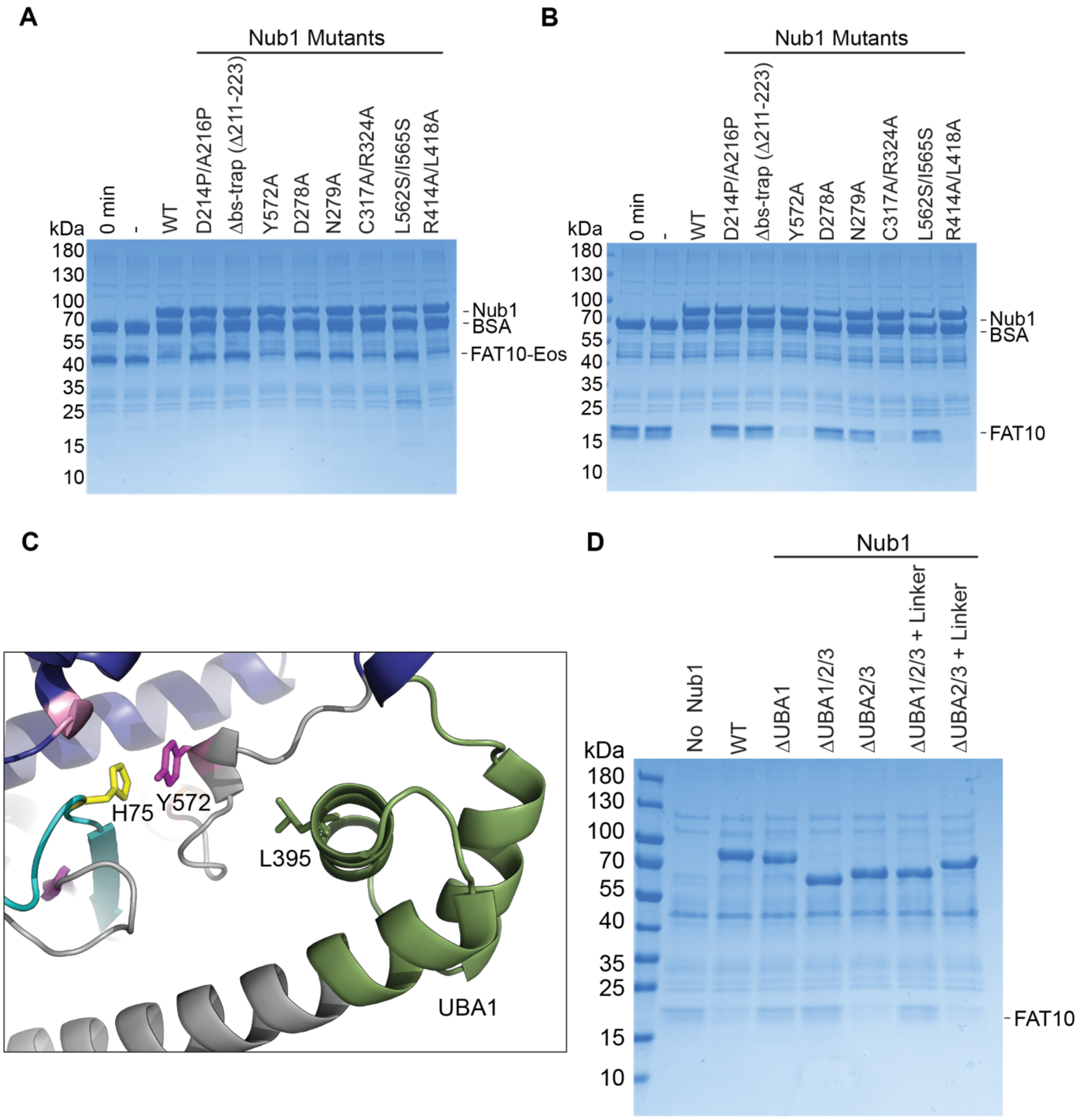

Supplementary Figure 7: NUB1's UBA1 domain and BS-Linker are critical for FAT10 degradation by the *hs*26S proteasome. A) Coomassie-stained SDS-PAGE gel analysis of the endpoints for the degradation of FAT10-Eos (1 μM) by the *hs*26S proteasome (100 nM) in the presence of various NUB1 mutants (5 μM). **B)** Assay as in A), but using FAT10 (10 μM). **C)** Structural model of the NUB1/FAT10 complex, with FAT10 in cyan and NUB1 in blue, grey, and green, showing the direct interaction of FAT10's H75 with NUB1's Y572, which appears to be stabilized by L395 and a helix in NUB1's UBA1 domain. This interaction explains the FAT10-binding-induced protection of NUB1's UBA1 domain in HDX-MS experiments. **D)** Coomassie-stained SDS-PAGE analysis of FAT10 degradation (5 μM) by *hs*26S proteasome (100 nM) in the presence of various truncation mutants of NUB1 (10 μM), showing that NUB1's UBA2 and UBA3 domains are dispensable, while the UBA1 domain is essential for degradation. For complete gel images see Suppl. Fig. 13B.

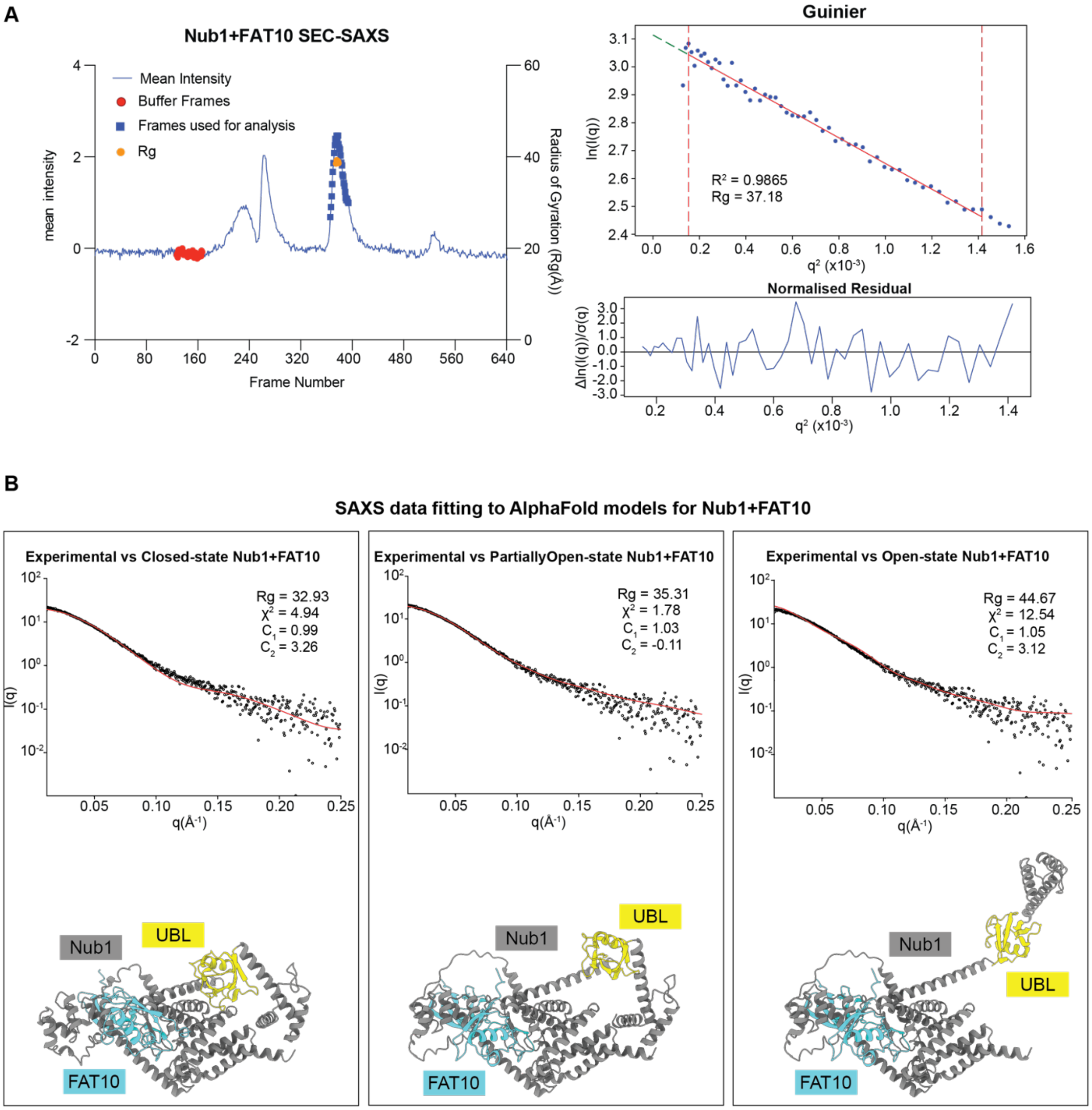

Supplementary Figure 8: Comparison of solution-state SAXS measurements with AlphaFold models of NUB1 bound to FAT10. A) Left: SEC-SAXS trace for NUB1 in complex with FAT10, with the frames used for buffer subtraction and analyses indicated by red dots and blue squares, respectively. Right: Guinier plot for the buffer-subtracted averaged SAXS frames of NUB1+FAT10. **B)** Fit of experimental SAXS data to the theoretical scattering curves for NUB1+FAT10 in the closed, partially open, and open states, using FOXS [66,67]. AlphaFold only generated a closed-state model, and the models for the partially open and open states were created by merging the corresponding models for the isolated NUB1 with NUB1-bound FAT10.

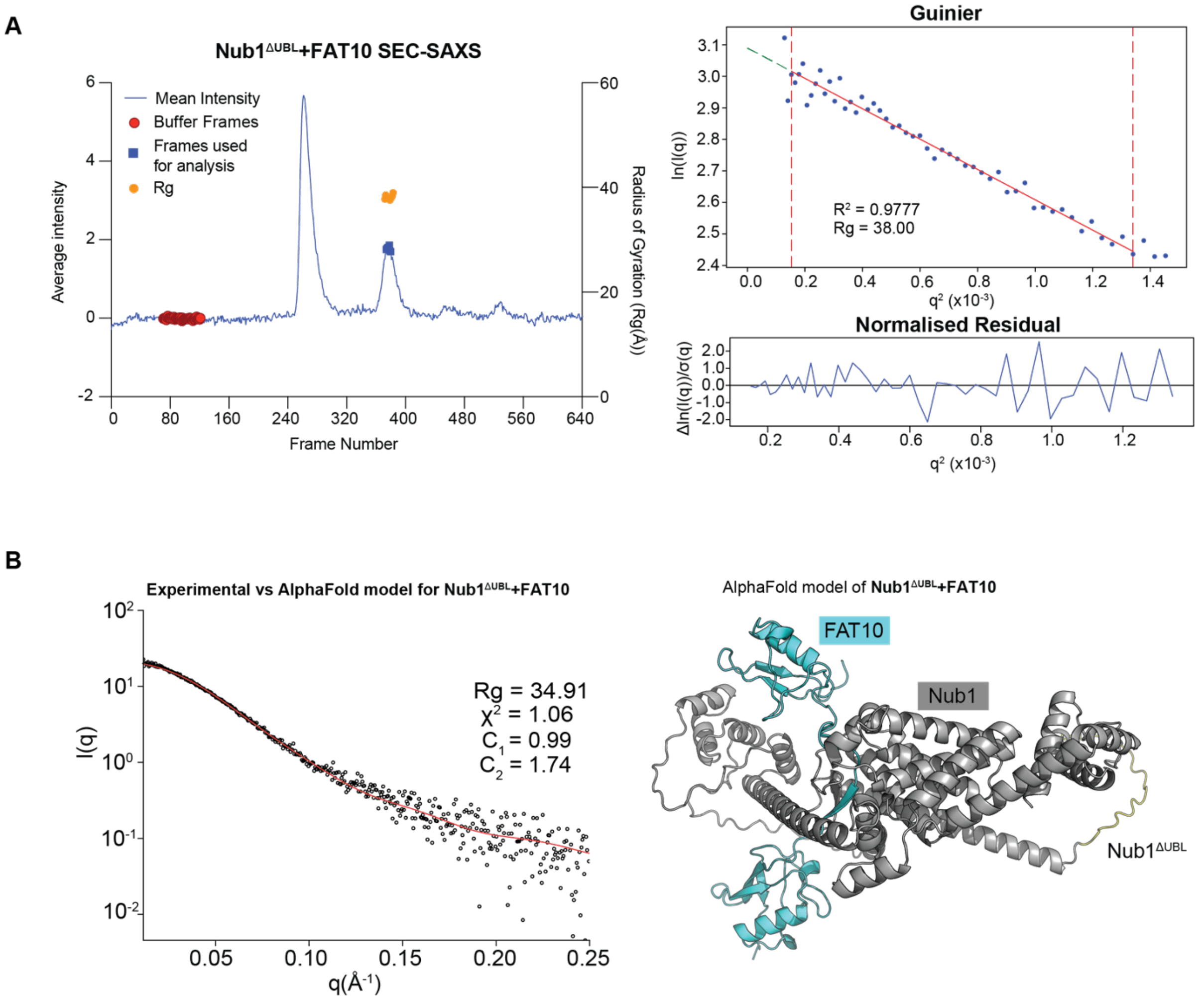

Supplementary Figure 9: Comparison of solution-state SAXS measurements with AlphaFold models of NUB1$^{\Delta UBL}$ bound to FAT10. A) Left: SEC-SAXS trace for NUB1$^{\Delta UBL}$+FAT10 with the frames used for buffer subtraction and analyses indicated by red dots and blue squares, respectively. Right: Guinier plot for the buffer-subtracted averaged SAXS frames of NUB1$^{\Delta UBL}$+FAT10. **B)** Fit of experimental SAXS data to AlphaFold-generated model for NUB1$^{\Delta UBL}$+FAT10, using FOXS [66,67].

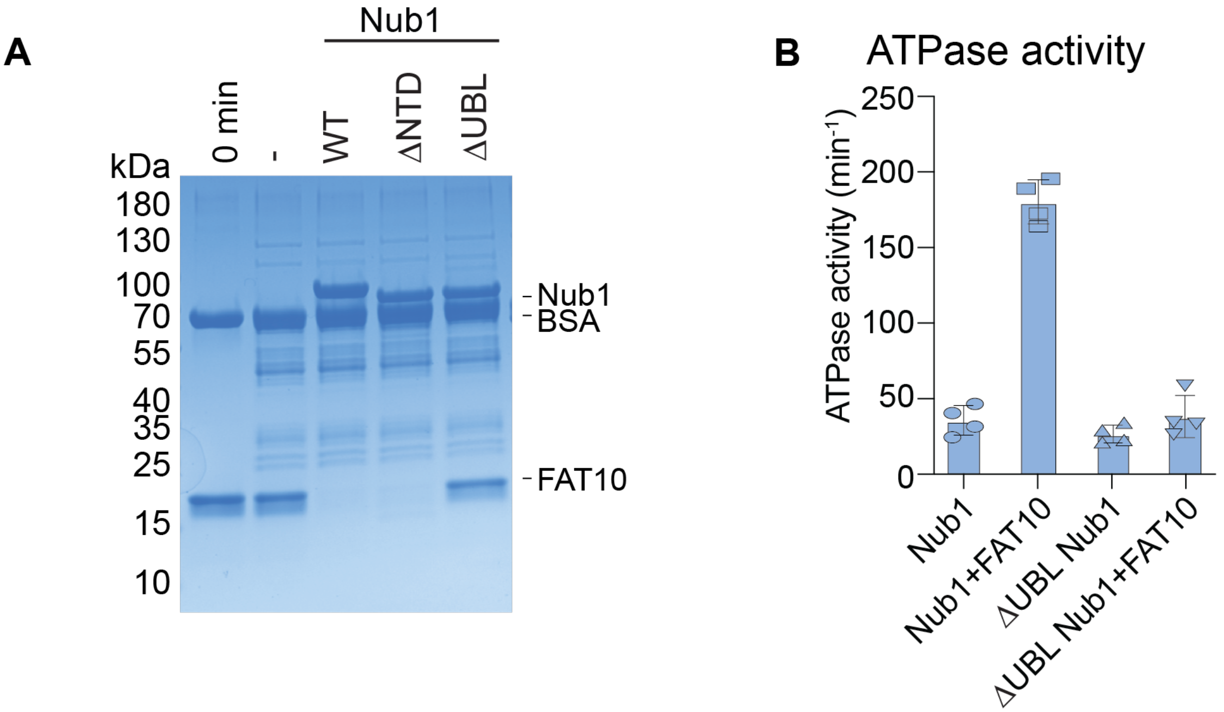

Supplementary Figure 10: NUB1's UBL domain is critical for FAT10 delivering to the proteasome. A) Coomassie-stained SDS-PAGE gel analysis of the endpoints for the degradation of FAT10 (10 µM) by *hs*26S proteasome (100 nM) in the presence of various NUB1 truncation mutants (2.5 µM). For complete gel image see Suppl. Fig. 13C. **B)** ATPase activity of *hs*26S proteasome in the absence and presence of Fat10, NUB1, or NUB1$^{\Delta UBL}$. Stimulation of ATP hydrolysis depends on NUB1's UBL domain for FAT10 delivery and the engagement of the FAT10 with the ATPase motor to shift the proteasome conformation from engagement-competent to processing states. Data show the mean +/- SD for n = 4 technical replicates.

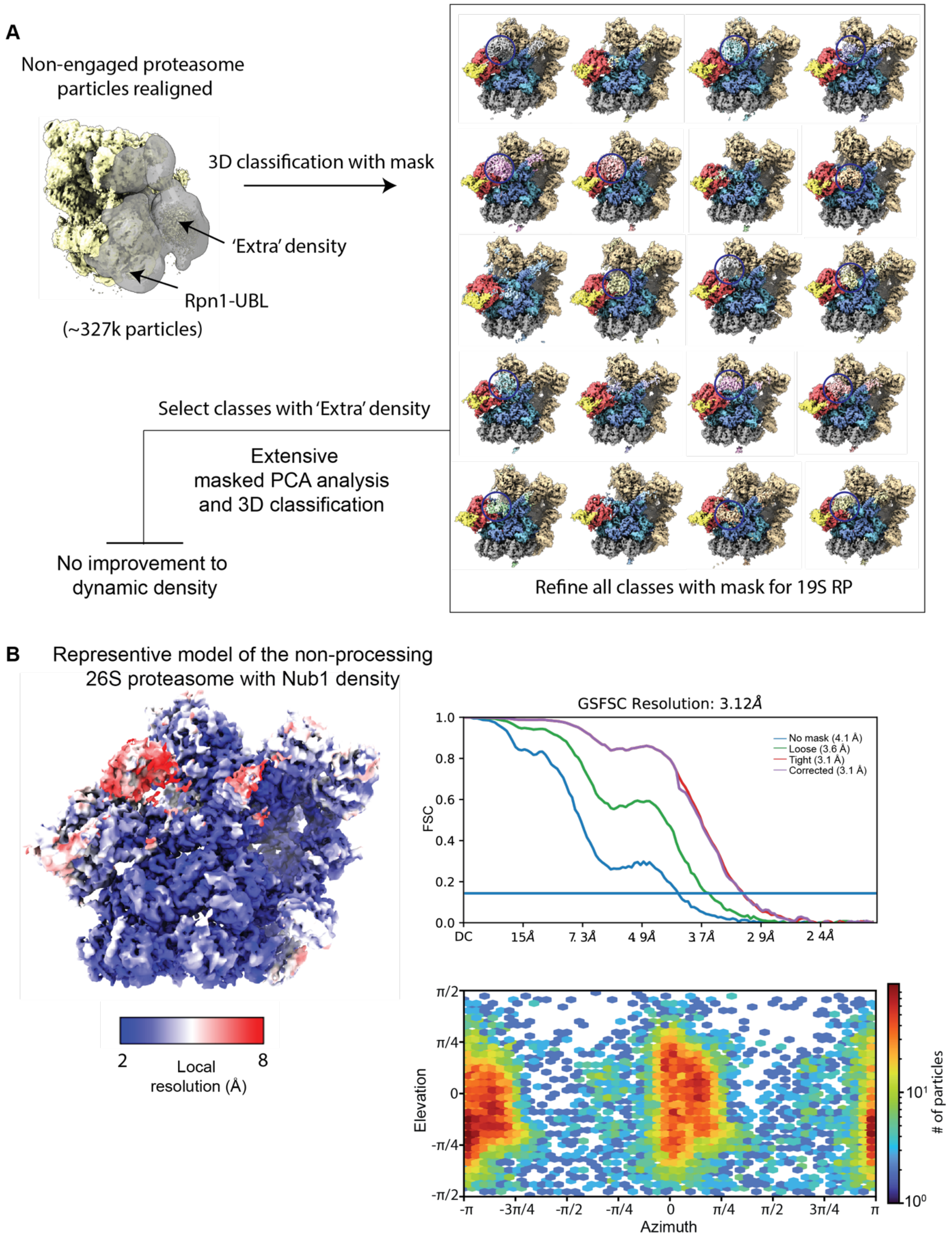

Supplementary Figure 11: Continued Cryo-EM processing for the 30 s data set, focusing on the extra density connected to NUB1's UBL domain. A) Extensive attempts were made to resolve the additional density attached to NUB1's UBL domain. The 'best' results for

representation were low-resolution amorphous masses for the extra density, which likely contains NUB1's core domain bound to an unfolded FAT10-Eos molecule. This suggests that NUB1 is dynamic with respect to the relative orientation of its core and UBA domains and is moving continually relative to its UBL domain, independent of the 26S proteasome. Focused refinements before and after particle subtractions of the 19S RP signal for extra density also failed to generate any interpretable models, likely to due to weak signal compared to the large 26S proteasome, the continuous motions within NUB1 itself, and the unfolded FAT10 molecule which in intrinsically dynamic. **B)** Representative model for the non-processing proteasome with bound NUB1/FAT10-Eos. Shown also are the Gold-Standard FSC plot and distribution of particle orientations.

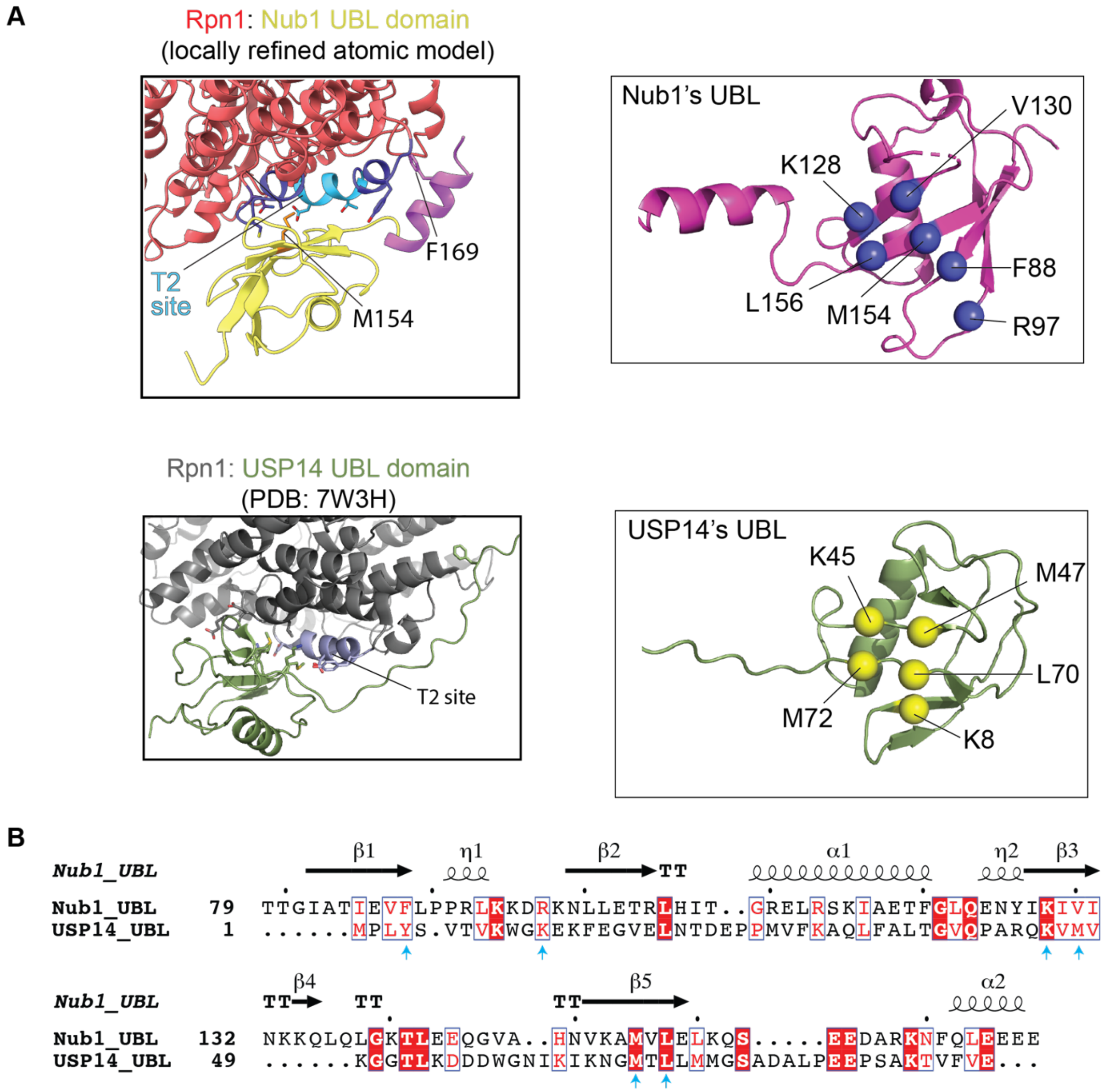

Supplementary Figure 12: Comparison of NUB1's UBL domain and USP14's UBL domain bound to the T2 site of Rpn1. A) Left top and bottom: Structures of NUB1's UBL domain (yellow) and USP14's UBL domain (green) bound to Rpn1's T2 site (light and slate blue). The representations highlight how both UBL domains bind the T2 site of Rpn1 through their beta sheet surface, which is structurally equivalent to the ubiquitin I44 hydrophobic patch as a common site for protein-protein interactions. Both UBL domains also use part of a C-terminally located linker, which connects the UBL domain to the rest of the respective protein, for additional interactions with Rpn1, yet at distinct sites. The positions of NUB1's UBL domain and USP14's UBL domain slightly vary with respect to Rpn1, potentially due differences in proteasome conformations and transient interactions formed between Rpn1 and the linkers. **B)** Sequence alignment of the UBL domains of human NUB1 and human USP14, generated with Clustal Omega and ESPript [68] (https://espript.ibcp.fr).

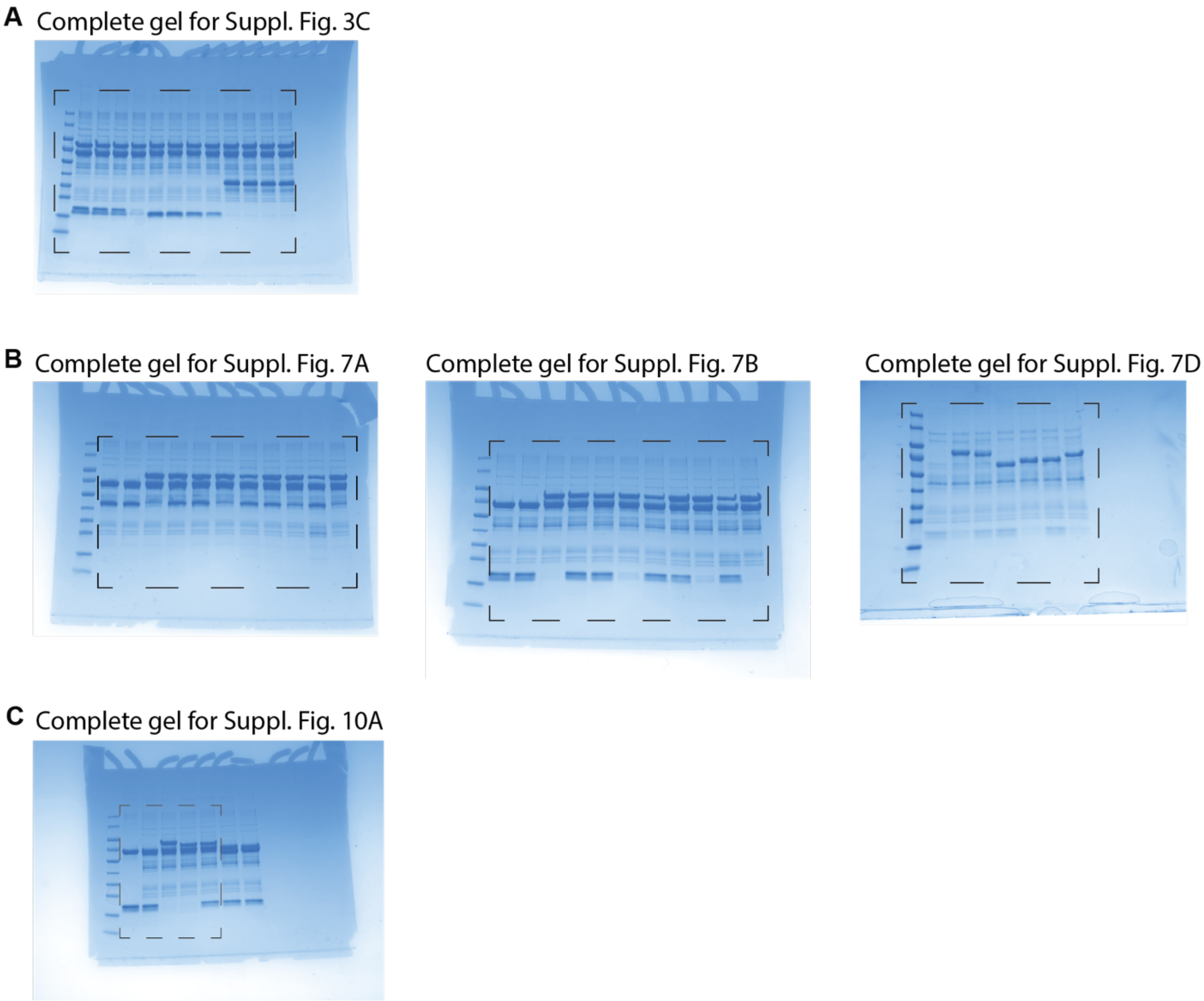

Supplementary Figure 13: Source data showing the complete Coomassie-stained gels for the gel images depicted in Suppl. Fig. 3C (**A**), Suppl. Fig. 7A, B, and D (**B** left, middle, and right), and Suppl. Fig. 10A (**C**).

	Peptides	Coverage	Average length	SD	Average Redundancy
Nub1	573	98.7%	13.1	6.4	12.4
FAT10	189	90.3%	16.0	7.3	18.3

Supplementary Table 1: Peptide-coverage statistics for HDX-MS experiments of FAT10 and NUB1. Peptides for FAT10 and NUB1 were only kept if found in both unbound and bound samples. Data were generated using HDExaminer 3.
