# Supplementary material for: NUB1 traps unfolded FAT10 for ubiquitin-independent degradation by the 26S proteasome
Source: Nat Struct Mol Biol. 2025 Apr 11;32(9):1752–65. doi: 10.1038/s41594-025-01527-3 (PMC12285911; doi:10.1038/s41594-025-01527-3)
Supplement: Supplementary file 2 — Reporting Summary [file 41594_2025_1527_MOESM2_ESM.pdf]

nature portfolio

Corresponding author(s): Andreas Martin

Last updated by author(s): 12/18/2024

Reporting Summary

Nature Portfolio wishes to improve the reproducibility of the work that we publish. This form provides structure for consistency and transparency in reporting. For further information on Nature Portfolio policies, see our Editorial Policies and the Editorial Policy Checklist.

Statistics

For all statistical analyses, confirm that the following items are present in the figure legend, table legend, main text, or Methods section.

n/a	Confirmed	
☐	☒	The exact sample size (*n*) for each experimental group/condition, given as a discrete number and unit of measurement
☐	☒	A statement on whether measurements were taken from distinct samples or whether the same sample was measured repeatedly
☐	☒	The statistical test(s) used AND whether they are one- or two-sided<br>*Only common tests should be described solely by name; describe more complex techniques in the Methods section.*
☒	☐	A description of all covariates tested
☒	☐	A description of any assumptions or corrections, such as tests of normality and adjustment for multiple comparisons
☐	☒	A full description of the statistical parameters including central tendency (e.g. means) or other basic estimates (e.g. regression coefficient) AND variation (e.g. standard deviation) or associated estimates of uncertainty (e.g. confidence intervals)
☐	☒	For null hypothesis testing, the test statistic (e.g. *F*, *t*, *r*) with confidence intervals, effect sizes, degrees of freedom and *P* value noted<br>*Give P values as exact values whenever suitable.*
☒	☐	For Bayesian analysis, information on the choice of priors and Markov chain Monte Carlo settings
☒	☐	For hierarchical and complex designs, identification of the appropriate level for tests and full reporting of outcomes
☒	☐	Estimates of effect sizes (e.g. Cohen's *d*, Pearson's *r*), indicating how they were calculated

Our web collection on statistics for biologists contains articles on many of the points above.

Software and code

Policy information about availability of computer code

Data collection	MARS by ClarioStar was used for collecting fluorescence time traces. Xcalibur4.1 (Thermo Scientific) was used for mass-spec data collection.<br>Serial EM 4.1 was used for cryo-EM data collection.<br>SAXS data were collected on a Pilatus3 2M pixel array detector.
Data analysis	HDX-Mass-spec data analysis was performed using Byonic (Protein Metrics) and HDExaminer 3.2 (Sierra Analytics). Jupiter notebooks using python was used for generating Woods plots and PAE plots for AlphaFold models. CollabFold was used to generate all AlphaFold structural models for Nub1 and the Nub1/FAT10 complex.<br>For cryo-EM structural data analyses and visualization we used Phenix v1.20.1, Coot 1.0, CryoSparc 4.0, UCSF Chimera v1.14, ChimeraX-1.7.1, and Pymol 2.5.2. All kinetic analyses and statistics were done in Graphpad 10.2.3. (Prism).<br>SAXS data were analyzed using RAW and FoXS

For manuscripts utilizing custom algorithms or software that are central to the research but not yet described in published literature, software must be made available to editors and reviewers. We strongly encourage code deposition in a community repository (e.g. GitHub). See the Nature Portfolio guidelines for submitting code & software for further information.

Data

Policy information about availability of data

All manuscripts must include a data availability statement. This statement should provide the following information, where applicable:

- Accession codes, unique identifiers, or web links for publicly available datasets
- A description of any restrictions on data availability
- For clinical datasets or third party data, please ensure that the statement adheres to our policy

All data generated or analyzed during this study are included in this manuscript and the Supplementary materials. Structural data are available in the Electron Microscopy Databank and the RCSB Protein Databank (EMDB-42506 and PDB ID 8USB for the non-processing 26S proteasome, EMDB-42507 and PDB ID 8USC for the substrate-processing 26S proteasome at 30 s after substrate addition, and EMDB-42508 and PDB ID 8USD for focused refinement of the proteasomal Rpn1 subunit with bound UBL domain of NUB1).
SEC-SAXS data for Nub1 can be found at https://simplescattering.com/ with the code: XSPKOJGM.
Any additional information required to reanalyze the data reported in this paper is available from the lead contact upon request.

Research involving human participants, their data, or biological material

Policy information about studies with human participants or human data. See also policy information about sex, gender (identity/presentation), and sexual orientation and race, ethnicity and racism.

Reporting on sex and gender	NA
Reporting on race, ethnicity, or other socially relevant groupings	NA
Population characteristics	NA
Recruitment	NA
Ethics oversight	NA

Note that full information on the approval of the study protocol must also be provided in the manuscript.

Field-specific reporting

Please select the one below that is the best fit for your research. If you are not sure, read the appropriate sections before making your selection.

☒ Life sciences ☐ Behavioural & social sciences ☐ Ecological, evolutionary & environmental sciences

For a reference copy of the document with all sections, see nature.com/documents/nr-reporting-summary-flat.pdf

Life sciences study design

All studies must disclose on these points even when the disclosure is negative.

Sample size	Biochemical experiments were performed in bulk measurements and with at least 3 technical replicates, and sample-size considerations therefore do not apply. For cryo-EM structure determination, 20,565 and 19,128 corrected micrographs were collected and a smaller subset of ~ 50,000 particles was used for ab-initio reconstructions.
Data exclusions	No data exclusions
Replication	All measurements were done with at least 3 technical replicates. All measurements could be replicated.
Randomization	NA for our in vitro biochemical and cryo-EM structural studies, as these are ensemble techniques
Blinding	NA for in vitro biochemical and cryo-EM structural studies, which analyze large ensembles of molecules

Reporting for specific materials, systems and methods

We require information from authors about some types of materials, experimental systems and methods used in many studies. Here, indicate whether each material, system or method listed is relevant to your study. If you are not sure if a list item applies to your research, read the appropriate section before selecting a response.

Materials & experimental systems

n/a	Involved in the study
☒	☐ Antibodies
☐	☒ Eukaryotic cell lines
☒	☐ Palaeontology and archaeology
☒	☐ Animals and other organisms
☒	☐ Clinical data
☒	☐ Dual use research of concern
☒	☐ Plants

Methods

n/a	Involved in the study
☒	☐ ChIP-seq
☒	☐ Flow cytometry
☒	☐ MRI-based neuroimaging

Eukaryotic cell lines

Policy information about cell lines and Sex and Gender in Research

Cell line source(s)	HEK293 cells with HTBH-tagged human 26S proteasomes were a gift from Lan Huang (UC Irvine)
Authentication	The cell line was authenticated by the successful affinity purification of human proteasomes
Mycoplasma contamination	The cell line was not tested for Mycoplasma contamination
Commonly misidentified lines (See ICLAC register)	No commonly misidentified cell lines were used in this study

Plants

Seed stocks	NA
Novel plant genotypes	NA
Authentication	NA
