# Supplementary figures and images for: NUB1 traps unfolded FAT10 for ubiquitin-independent degradation by the 26S proteasome
Source: Nat Struct Mol Biol. 2025 Apr 11;32(9):1752–65. doi: 10.1038/s41594-025-01527-3 (PMC12285911; doi:10.1038/s41594-025-01527-3)

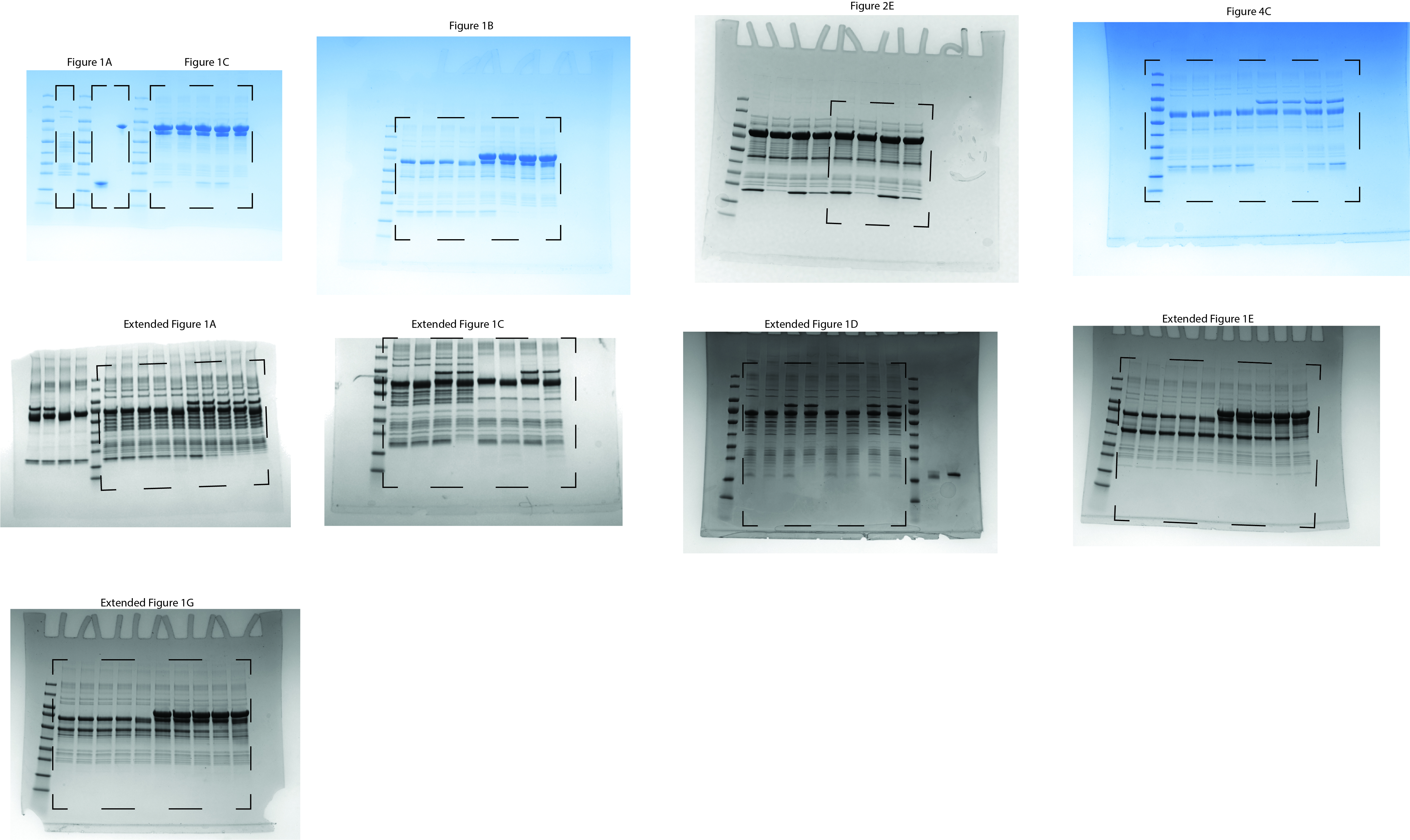

Supplement: Supplementary file 10 — Images of all uncropped gels. [file 41594_2025_1527_MOESM10_ESM.jpg]
